# Human effector CD8^+^ T cells with an activated and exhausted-like phenotype control tumour growth *in vivo* in a humanized tumour model

**DOI:** 10.1016/j.ebiom.2024.105240

**Published:** 2024-07-09

**Authors:** Juliane Mietz, Meike Kaulfuss, Lukas Egli, Lennart Opitz, Christian Münz, Obinna Chijioke

**Affiliations:** a Cellular Immunotherapy, Institute of Experimental Immunology, University of Zürich, Zürich, Switzerland; b Functional Genomics Center Zürich, University of Zürich/ETH Zürich, Zürich, Switzerland; c Viral Immunobiology, Institute of Experimental Immunology, University of Zürich, Zürich, Switzerland; d Institute of Medical Genetics and Pathology, University Hospital Basel, Basel, Switzerland

**Keywords:** Human cancer immunology, Humanized cancer models, Phenotypic markers, T cells, Exhaustion, Adoptive cell therapy

## Abstract

**Background:**

Humanized tumour models could be particularly valuable for cancer immunotherapy research, as they may better reflect human-specific aspects of the interfaces between tumour and immune system of human cancer. However, endogenous antitumour immunity in humanized models is still largely undefined.

**Methods:**

We established an autologous humanized mouse tumour model by using NSG mice reconstituted with human immune cells from hematopoietic progenitors and tumours generated from transformed autologous human B cells. We demonstrate growth of solid lymphoid tumours after subcutaneous implantation, infiltration by endogenous human immune cells and immunocompetence of the model.

**Findings:**

We found human T cell subsets described in human cancer, including progenitor exhausted (T_pex_), terminally exhausted (T_ex-term_) and tissue-resident (T_RM_) cells in tumour-bearing humanized mice with accumulation of T_ex-term_ and T_RM_ in the tumour. In addition, we identified tumour-reactive CD8^+^ T cells through expression of CD137. This subpopulation of de novo arising human CD137^+^ CD8^+^ T cells displayed a highly proliferative, fully activated effector and exhausted-like phenotype with enhanced expression of activation and exhaustion markers like PD-1, CD39, CD160, TIM-3, TIGIT and TOX, the senescence marker CD57 (*B3GAT1*) and cytolytic effector molecules such as *PRF1*, *GZMH* and *NKG7*. Moreover, these CD137^+^ CD8^+^ T cells exhibited tumour-specific clonal expansion and presented signature overlap with tumour-reactive CD8^+^ T cells described in human cancer. We demonstrate superior anticancer activity of this activated and exhausted-like human CD8^+^ T cell subset by adoptive transfer experiments using recipients bearing autologous human tumours. Mice adoptively transferred with CD137^+^ CD8^+^ T cells showed reduced tumour growth and higher CD8^+^ T cell tumour infiltration, correlating with control of human tumours.

**Interpretation:**

We established an immunocompetent humanized tumour model, providing a tool for immunotherapy research and defined effective anticancer activity of human effector CD8^+^ T cells with an activated and exhausted-like phenotype, supporting clinical exploration of such cells in adoptive T cell therapies.

**Funding:**

10.13039/501100013362Swiss Cancer Research foundation.

**Research in context**

**Evidence before this study**

Antitumour immune responses and outcome of immunotherapeutic interventions are not always consistent between mouse models of cancer and data available in humans. This may be due to species-specific differences, therefore models with a potential for better translatability are needed, such as humanized mouse models. However, there is limited data on human antitumour T cell immunity in humanized mice.

**Added value of this study**

In this study, we established an immunocompetent humanized tumour model that recapitulates hallmarks of human antitumour T cell responses, offering the possibility for further translational investigation of the interface between human tumours and endogenous anticancer immunity. Furthermore, using functional *in vitro* assays and adoptive transfer, our study demonstrates the key importance of human effector CD8^+^ T cells with an activated and exhausted-like phenotype in the antitumour immune response.

**Implications of all the available evidence**

The autologous humanized tumour model provided in this study can serve as a tool to elucidate human-specific immune features. By bridging a gap between syngeneic mouse tumour models and human-specific antitumour immune responses, the model may help open up avenues for greater translatability of preclinical data. Our findings suggest that exhausted-like effector CD8^+^ T cells can be harnessed for clinical development of adoptive T cell therapies.

## Introduction

The characterization of phenotypic traits that can faithfully identify tumour-specific CD8^+^ T cells with functional anticancer activity is an area of intense research. Recent studies have shown tumour-reactive CD8^+^ T cells to be rare in patients with various cancers and thus challenging to explore.^1^^,^^2^ Preclinical studies in mice have shown that CD8^+^ T cells displaying an exhausted-like phenotype have decreased effector function.^3^ Reinvigoration of exhausted antitumour T cells is thought to be a key component of response to immune checkpoint blockade. Nevertheless, studies in individuals with cancer have correlated exhausted-like phenotypic states of CD8^+^ T cells with tumour reactivity and clinical benefit.^4^^,^^5^

Undoubtedly, tumour models using inbred and transgenic mice have contributed substantially to our understanding of tumour immunobiology and have been fundamental to the development of cancer immunotherapies that are now used on a routine basis in the clinic.^6^^,^^7^ However, human clinical studies^8^ have shown that the outcome of cancer immunotherapies can significantly diverge from those of mouse models of cancer.^9^ This might be due to a relative lack of genetic diversity of mouse models compared to humans^10^ but also to differences in aspects of gene expression networks,^11^ genomic organization^12^ and tissue structure of the immune system of mice and humans.^13^ Xenograft mouse models using adoptive transfer of human immune cells rely on immunodeficient hosts, precluding analysis of the impact of endogenous adaptive antitumour immunity. Furthermore, in most preclinical studies investigating novel immunotherapy targets, monoclonal T cells with a single antigen specificity are explored^14^^,^^15^ while polyclonal T cell responses might be a biomarker for favourable patient outcome to immunotherapy^16^ and thus be more informative.

Complementing existing mouse tumour models, the use of mice with reconstituted human immune system components (HIS mice) holds hitherto underexplored translational potential to advance human cancer immunology.^17^ Yet, endogenous antitumour immunity in these models has remained largely undefined, mainly due to allogeneic reactivity of the reconstituted human T cells against allogeneic tumours,^18^ making a distinction of alloreactivity from tumour-specific immune responses challenging.

In this study, we demonstrate that reconstitution of human immune system components across independent donors can mount endogenous antitumour T cell responses to autologous human tumour challenge in HIS mice. A polyclonal population of human effector CD8^+^ T cells with an activated and exhausted-like phenotype that arises de novo in tumour-bearing HIS mice, resembling exhausted tumour-reactive lymphocytes found in patients with cancer, is able to control autologous human tumour growth *in vivo*. Thus, we provide a tool to investigate human antitumour T cell immunity and direct evidence of clinically relevant anticancer activity of expanded human tumour-reactive effector CD8^+^ T cells with an activated and exhausted-like, non-progenitor-like phenotype.

## Methods

### HIS & NSG mice

NSG (NOD.Cg-Prkdc<scid>Il2rg<tm1Wjl>/SzJ (#005557)) mice were purchased from The Jackson Laboratory and bred and housed under specific pathogen-free conditions at the Laboratory Animal Services Center (LASC) Zurich, University of Zurich. Mice were housed under a 12-h light/12-h dark cycle at temperatures from 21 to 24 °C with 35–70% humidity with constant access to water and food. Crinkles, tissues, and gnawing sticks were provided as cage enrichment. Up to 5 mice were housed per cage. Per cage, mice of different treatment groups were mixed to reduce cage-related confounding factors. Male and female mice were used for experiments at the age of 3–5 months. Mice were transferred at least 7 days before experiments started to the location where experiments took place. To generate HIS mice, new-born pups (1–5 days old) were irradiated (1 Gy) and intrahepatically injected with 0.2 × 10^6^ human CD34^+^ hematopoietic progenitor cells derived from human foetal liver (Advanced Bioscience Resources, USA). CD34^+^ cells were isolated by MACS technology (Miltenyi, Cat. 130-046-703) following the manufacturer's instructions. At 3 months of age, reconstitution of human immune cells was assessed in PBMC from tail vein blood by flow cytometry.^19^ Reconstitution analysis included staining for human CD45, CD3, CD4, CD8, CD19, NKp46 and HLA-DR. Only mice with a sufficiently high frequency of human CD45 (>20% of leukocytes) were included in the experiments. Mice that showed Graft-versus-Host-disease (GvHD)-like symptoms like fur loss, scratching or crusted skin were excluded from experiments. Mice were assigned to treatment groups based on the reconstitution frequency (%CD45 and %CD3) to generate equally reconstituted groups. If possible, female and male mice were equally distributed to treatment groups. Blinding was performed during data acquisition. A total of 128 mice (79 HIS mice, 49 NSG mice) were used for this study. Generally, the experimental unit was single animal. All animal experiments were performed according to approved licenses by the veterinary office of the canton of Zurich, Switzerland (ZH049/20 and ZH067/2023).

### LCL generation and tumour model

LCL were generated from CD19^+^ B cells derived from the same autologous human foetal liver tissue from which the CD34^+^ cells for reconstitution of the human immune system in the same experiment were derived. CD19^+^ cells were isolated from the HFL tissue's CD34^-^ fraction by MACS technology (Miltenyi, Cat. 130-050-301) according to the manufacturer's instructions. 0.25 × 10^6^ CD19^+^ human B cells were transformed by infection with Epstein–Barr virus (EBV B95-8; produced as previously described^19^) at an MOI of 0.1–0.15 and cultured in RPMI/10% FCS/1% Penicillin/Streptomycin.

LCL tumours were implanted subcutaneously into the left flank under isoflurane narcosis. For generation and phenotyping of tumour-reactive T cells, 5 × 10^6^ autologous LCL tumour cells were resuspended in PBS and right before injection mixed in a 1:1 V/V ratio with Corning® Matrigel® Growth Factor Reduced (GFR) Basement Membrane Matrix (Milian, Cat. 354230). For phenotyping of tumour-reactive human T cells in HIS mice, a total of 23 mice was used in 6 independent experiments (n = 3–4). To phenotype human T cells from naïve HIS mice, a total of 10 mice from 5 independent experiments was used (n = 1–4). To characterize growth behaviour of LCL tumours in HIS and NSG mice, a total of 18 mice was used (9 HIS mice, 9 NSG mice), which were s.c. injected with 2 × 10^6^ LCL (n = 3), 5 × 10^6^ LCL (n = 3) or 10 × 10^6^ LCL (n = 3).

### Adoptive cell transfer experiments

The primary outcome measure of adoptive cell transfer experiments was tumour volume. Sample sizes were calculated using G∗Power software^20^ (one-tailed t-test, effect size (signal-to-noise-ratio) = 2.5, α error probability = 0.05, power = 0.8), which resulted in a group size of 3 mice per group. Other outcome measures assessed were immune cell phenotyping in tumour, spleen and peripheral blood.

For adoptive cell transfer experiments with HIS recipient mice, 2 × 10^6^ LCL were injected s.c. in a 1:1 V/V mix with Matrigel and three days after tumour injection, 2 × 10^6^ T cells were adoptively transferred by tail vein injection. Transferred T cell subsets were *ex vivo* expanded autologous CD137^+^ CD8^+^, CD137^−^ CD8^+^ or CD137^−^PD-1^−^ CD8^+^ T cells and were compared against mice that did not receive T cells (tumour only). A total of 37 mice was used, of which 14 mice received CD137^+^ CD8^+^ T cells in 3 independent experiments (n = 3–7), 5 mice received CD137^−^ CD8^+^ T cells in one experiment (n = 5), 6 mice received CD137^−^PD-1^−^ CD8^+^ T cells in 2 independent experiments (n = 3) and 12 mice received no T cells (tumour only) in 3 independent experiments (n = 3–6). One mouse from the CD137^+^ group and 2 mice from the CD137^−^ group were euthanized early and excluded from the analysis due to the development of an ulceration. Treatment groups differed between experiments if the respective cell population could not be expanded to sufficient numbers or not enough humanized mice were available for all groups.

For adoptive cell transfer experiments with NSG recipient mice, 2 × 10^6^ LCL were injected s.c. in a 1:1 V/V mix with Matrigel and three days after tumour injection, 10 × 10^6^ T cells were adoptively transferred by tail vein injection. Transferred T cell subsets were *ex vivo* expanded autologous CD137^+^ CD8^+^, CD137^−^PD-1^−^ CD8^+^ T cells or bulk CD8^+^ T cells form naïve donor mice and were compared against mice that did not receive T cells (tumour only). A total of 40 mice was used, of which 13 mice received CD137^+^ CD8^+^ T cells in 3 independent experiments (n = 4–5), 8 mice received CD137^−^PD-1^−^ CD8^+^ T cells in 2 independent experiments (n = 4), 6 mice received bulk CD8^+^ T cells (naïve donors) in 2 independent experiments (n = 3) and 13 mice received no T cells (tumour only) in 3 independent experiments (n = 4–5). One mouse from the tumour only group was euthanized early and excluded from the analysis due to the development of an ulceration. Treatment groups differed between experiments if the respective cell population could not be expanded to sufficient numbers.

Tumour size was monitored by calipering (3×/week or daily). Tumour volume was calculated as volume [mm^3^] = length [mm] x width^2^ [mm^2^] x 0.52. General health was monitored by weighing and health parameter scoring (3×/week or daily, according to animal license). If mice showed signs of pain or distress, they were treated with paracetamol and carefully monitored and euthanized, if necessary. PBMC composition and expansion of adoptively transferred T cells were monitored by weekly tail vein bleeding and flow cytometric analysis. White blood cell (WBC) counts were measured from blood with an automatic cell counting machine (DxH 500, Beckman Coulter). Animals were euthanized when they met pre-defined termination criteria defined in the animal license, including weight loss >20%, body condition score = 1, tumour diameter >15 mm, tumour ulceration, severe hunching, apathic behaviour, dyspnoea, paralysis, severe paleness or severe GvHD-like symptoms.

### T cell isolation and expansion

Spleens from LCL tumour-bearing and naïve HIS mice were harvested 16–18 days after s.c. tumour injection. Splenocytes were labelled with fluorescently labelled antibodies and sorted by fluorescent activated cell sorting (FACS). CD137^+^, CD137^−^ and CD137^−^PD-1^−^ CD8^+^ T cells were sorted from tumour-bearing HIS mice and bulk CD8^+^ T cells were isolated from naïve HIS mice. Sorted T cells were expanded by a rapid expansion protocol (REP), as described elsewhere.^21^ REP medium consisted of a 1:1 mix of RPMI (Gibco, Cat. 7001612) and X-Vivo (Lonza, Cat. BE02-060F) containing 10% FCS (Biochrome, Cat. S0615-500 ML) and 1% Penicillin/Streptomycin (Thermo Fisher, Cat. 7001592). After sorting, T cells were cultured in REP medium with 30 ng/μl OKT3 (Miltenyi, Cat. 130-093-387), 3000 IU/ml IL-2 (Peprotech, Cat. 200-02) and a 200-fold excess of irradiated (50 Gy) allogeneic PBMC derived from 3 healthy donors at a total cell density of 5 × 10^6^ cells/ml. Starting at day 7, cells were adjusted with REP medium to 0.5–1 x 10^6^ cells/ml and fresh IL-2 (3000 IU/ml) was added every second day. Cells were expanded for a total of 14–16 days until use for further experiments.

### Cell isolation from organs

For reconstitution analysis and weekly bleeding, 100–150 μl of tail vein blood was collected into EDTA tubes (BD Microtainer, BD, Cat. 365975) and erythrocytes were lysed by incubation with lysis buffer. Leukocytes were stained for flow cytometric analysis. For PBMC isolation on the day of sacrifice, blood was harvested by heart puncture and erythrocytes were lysed with erythrocyte lysis buffer. For splenocyte isolation, spleens were removed and meshed through a 70 μm cell strainer in PBS and lymphocytes were isolated by Ficoll–Paque (GE Healthcare, 17-5442-03). Single cell suspensions were washed and stained for flow cytometric analysis. For tumour-infiltrating lymphocyte (TIL) isolation, tumours were removed from the flank and skin was removed from the tumours. Tumours were cut into small pieces with scissors and then incubated with digestion mix (DMEM (Life technologies, Cat. 7001566) containing 2% FCS (Biochrome, Cat. S0615-500 ML), 1 mg/ml Collagenase IV (Roche, Cat. 7002219), 10ug/ml DNase I (Roche, Cat. 7002221) and 1.2 mM CaCl2; approx. 1.5 ml/tumour, incubation for 45 min, 37 °C). The tumour digest was diluted with 20 ml RPMI or PBS and meshed over a 70 μm cell strainer. Single cell suspensions were washed and then stained for flow cytometric analysis.

### Flow cytometry

For surface staining, cells were washed in PBS and stained with an antibody master mix for 20 min at 4 °C in the dark. For intracellular staining, cells were first stained for surface antigens and fixable live/dead marker, then fixed and permeabilized using the BD Pharmingen™ Transcription Factor Buffer Set (BD, Cat. 562574) according to the manufacturer's instructions, then stained for intracellular or intranuclear antigens for 50–60 min. Cells were washed in PBS and acquired on a BD LSRFortessa™ or Cytek Aurora.

Antibodies for reconstitution analysis of HIS mice (all antibodies are anti-human): CD45-Pacific Blue (Biolegend, HI30, Cat. 304029), CD3-PE (Biolegend, UCHT1, Cat. 300408), CD4-APC-Cy7 (Biolegend, RPA-T4, Cat. 300518), CD8-PerCP (Biolegend, SK1, Cat. 344708), HLA-DR-FITC (Biolegend, L243, Cat. 307604), CD19-PE-Cy7 (Biolegend, HIB19, Cat. 302216), NKp46-APC (BD, 9-E2, Cat. 558051), Zombie Aqua™ Fixable Viability Kit (Biolegend, Cat. 423101).

Antibodies for flow cytometry-based sorting of T cells: CD3-FITC (Biolegend, UCHT1, Cat. 300406), CD4-APC-Cy7 (Biolegend, RPA-T4, Cat. 300518), CD8-BV650 (Biolegend, SK1, Cat. 344730), CD45-Pacific Blue (Biolegend, HI30, Cat. 304029), CD137-APC (Miltenyi, REA765, Cat. 130-110-901), PD-1-PE-Dazzle (Biolegend, EH12.2H7, Cat. 329940), Zombie Aqua™ Fixable Viability Kit (Biolegend, Cat. 423101).

Antibodies for phenotyping in Panel 1: CD45-Pacific Blue (Biolegend, HI30, Cat. 304029), CD3-PerCP-Cy5.5 (Biolegend, UCHT1, Cat. 300429), CD4-APC-Cy7 (Biolegend, RPA-T4, Cat. 300518), CD8-BV650 (Biolegend, SK1, Cat. 344730), CD45RA-BV785 (Biolegend, HI100, 304139), CD62L-FITC (BD, DREG-56, Cat. 555543), CD19-PE-Cy7 (Biolegend, HIB19, Cat. 302216), HLA-DR-BV605 (BD, g46-6, Cat. 562845), CD39-BV711 (Biolegend, A1, Cat. 328228), CD69-Alexa Fluor 700 (Biolegend, FN50, Cat. 310921), PD-1-PE-Dazzle (Biolegend, EH12.2H7, Cat. 329940), TCF1-PE (Biolegend, 7F11A10, Cat. 655207), CD137-APC (Miltenyi, REA765, Cat. 130-110-901), Zombie Aqua™ Fixable Viability Kit (Biolegend, Cat. 423101); in Panel 2: CD3-FITC (Biolegend, UCHT1, Cat. 300406), CD4-PE-Cy7 (Biolegend, RPA-T4, Cat. 300512), CD8-BV650 (Biolegend, SK1, Cat. 344730), CD45-BV711 (Biolegend, HI30, Cat. 304050), CD137-APC-Fire750 (Biolegend, 4B4-1, Cat. 309834), Granzyme B-APC-Alexa700 (Biolegend, QA16A02, Cat. 372222), Ki-67-BV605 (Biolegend, Ki-67, Cat. 350522), PD-1-PE-Dazzle (Biolegend, EH12.2H7, Cat. 329940), Perforin – BV421 (Biolegend, dG9, Cat. 308122), TCF1-PE (Biolegend, 7F11A10, Cat. 655207), TOX-APC (Miltenyi, REA473, Cat. 130-118-335), Zombie Aqua™ Fixable Viability Kit (Biolegend, Cat. 423101); in Panel 3: CD45-BUV395 (BD, HI30, Cat. 563792), CD3-Pacific Blue (Invitrogen, S4.1, Cat. MHCD0328), CD4-BUV495 (BD, SK3, Cat. 612936), CD8-BUV563 (BD, RPA-T8, Cat. 612914), CD19-PE-Cy5 (Biolegend, HIB19, Cat. 302210), CD45RA-BV785 (Biolegend, HI100, 304139), CD62L-BV605 (Biolegend, DREG-56, Cat. 304834), CD39-PE-Fire810 (Biolegend, A1, Cat. 328245), CD69-BUV805 (BD, FN50, Cat. 748763), CD137-APC (Miltenyi, REA765, Cat. 130-110-901), PD-1-PE-Dazzle (Biolegend, EH12.2H7, Cat. 329940), HLA-DR-FITC (Biolegend, LN3, Cat. 327006), TCF1-PE (Biolegend, 7F11A10, Cat. 655207), CD160-PerCP-Cy5.5 (Biolegend, BY55, Cat. 341210), LAG3-BV650 (Biolegend, 11C3C65, Cat. 369316), TIM3 – Alexa Fluor 700 (R&D Systems, Clone 344823, Cat. FAB2365N), Galectin-9 -PE-Cy7 (Biolegend, 9M1-3, Cat. 348916), TIGIT-APC-Cy7 (Biolegend, A15153G, Cat. 372734), Ki-67-BV510 (Biolegend, Ki-67, Cat. 350518), CD103-BV711 (Biolegend, Ber-ACT8, Cat. 350222), LIVE/DEAD™ Fixable Blue Dead Cell Stain Kit (Invitrogen, Cat. L23105).

The gating strategies for immunophenotyping by flow cytometry are provided in Supplemental Figure S2.

### Co-expression analysis

For co-expression analysis of flow cytometric data, cells were manually gated on live, singlet human CD8^+^ T cells. Cells were analysed using CRUSTY webtool,^22^ where clustering was performed using Phenograph algorithm and default settings for UMAP generation.

### IFN-γ ELISpot and TNFα ELISA

For IFN-γ ELISpot, ELISpot plates (Merck, Cat.MAIPN4550) were activated with 35% ethanol, washed and coated overnight with anti-IFN-**γ** coating antibody (mAb-1-D1K, Mabtech, Cat. 3420-3-1000). Plates were washed and T cells were co-cultured with autologous LCL in RPMI/10% FCS/1% Pen/Strep at an effector: target ratio of 5:1 for 16–24 h. After incubation, supernatant was harvested, stored at −20 °C and used for TNFα ELISA. Wells were washed and incubated for 2 h with biotinylated anti-IFN-**γ** detection antibody (mAb-7-B6-1-Biotin, Mabtech, Cat. 3420-6-250). Afterwards, wells were washed and incubated with Streptavidin-ALP (Mabtech, Cat. 3310-10) for 1 h. Wells were then washed again and spots were developed with filtered substrate solution BCIP/NBT-plus (Mabtech, Cat. 3650-10). When distinct spots were visible, the plate was washed and dried overnight. Plates were acquired with an ELISpot reader (AID classic, AID GmbH) and spots were automatically counted using AID ELISpot 8.0 software (AID GmbH).

TNFα ELISA was performed with the supernatant from T cell and LCL co-culture as described above using the human TNFα ELISA^BASIC^ kit (Mabtech, Cat. 3512-1H-6), following manufacturer's instructions. Plates were acquired using an ELISA reader (infinite M200Pro, Tecan) and analysed with the i-control 2.0 software (Tecan).

### Immunohistochemistry

Tumours were fixed in 4% paraformaldehyde and embedded in paraffin. Sample embedding, preparation and staining was performed by the Pathology department of the University Hospital Basel. Slides were acquired on an automated slide scanning brightfield microscope (Vectra 3, Akoya Biosciences) and quantified using InForm software (Akoya Biosciences).

### Bulk RNAseq and TCR profiling

RNA was extracted from 5 × 10^6^ T cells using the RNeasy® Mini Kit (Qiagen, Cat. 74106). RNA extraction was performed following the instructions from the “Purification of Total RNA from Animal Cells Using Spin Technology” protocol given in the RNeasy® Mini Handbook, including homogenization with QIAshredders (Qiagen, Cat. 79654) and on-column DNA digestion with the RNase-free DNase Set (Qiagen, Cat. 79254). All further steps were performed by the Functional Genomics Center Zurich (FGCZ). The quality of the isolated RNA was determined with a Qubit® (1.0) Fluorometer (Life Technologies, California, USA) and a Bioanalyzer 2100 (Agilent, Waldbronn, Germany). Only those samples with a 260 nm/280 nm ratio between 1.8 and 2.1 and a 28S/18S ratio within 1.5–2 were further processed. The TruSeq RNA Sample Prep Kit v2 (Illumina, Inc, California, USA) was used in the succeeding steps. Briefly, total RNA samples (600 ng) were poly A enriched and then reverse-transcribed into double-stranded cDNA. The cDNA samples were fragmented, end-repaired and polyadenylated before ligation of TruSeq adapters containing the index for multiplexing fragments containing TruSeq adapters on both ends were selectively enriched with PCR. The quality and quantity of the enriched libraries were validated using Qubit® (1.0) Fluorometer and the Caliper GX LabChip® GX (Caliper Life Sciences, Inc., USA). The product is a smear with an average fragment size of approximately 260 bp. The libraries were normalized to 10 nM in Tris-Cl 10 mM, pH8.5 with 0.1% Tween 20. After library quantification, libraries were prepared for loading accordingly to the NovaSeq workflow with the NovaSeq6000 Reagent Kit (Illumina, Catalog No. 20012865). Cluster generation and sequencing were performed on a NovaSeq6000 System with a run configuration of single end 100bp. RNAseq data analysis consisted of the following steps: The raw reads were first cleaned by removing adapter sequences, trimming low quality ends, and filtering reads with low quality (phred quality <20) using Fastp (Version 0.20).^23^ Sequence pseudo alignment of the resulting high-quality reads to the human reference genome (build GRCh38.p13 and GENCODE gene models release 37) and quantification of gene level expression was carried out using Kallisto (Version 0.46.1).^24^ Differential expression was computed using the generalized linear model implemented in the Bioconductor package DESeq2 (R version: 4.2.2, DESeq2 version: 1.38.1).^25^ Genes showing altered expression with adjusted (Benjamini and Hochberg method) p-value <0.05 were considered differentially expressed. Differentially expressed genes were explored using SUSHI.^26^ RNAseq expression data underwent preprocessing to remove duplicated genes. Subsequently, we utilized the CreateSeuratObject() function within the Bioconductor package Seurat (R version: 4.2.2, Seurat version: 5.0.1)^27^ to construct a Seurat object, facilitating downstream analyses. Normalization of the expression data was performed using the NormalizeData() function, with experimental group assignments explicitly indicated within the Seurat object. To delineate the gene signatures characterizing distinct T cell phenotypes, we curated pertinent signatures from previous studies. Genes absent in our expression data were systematically filtered out. Next, employing the AddModuleScore() function, we computed module scores for each gene signature across experimental groups and values were scaled for heatmap visualization. For the TCR analysis the read alignment to the Human genome was performed with STAR (v2.7.10b).^28^ TCR sequences (CDR3 of TRA, TRB, TRG and TRD) were reconstructed from bulk RNAseq data using TRUST4 (v1.0.10).^29^ Out-of-frame sequences were excluded from the analysis. TCR repertoire analysis was performed using the R package Immunarch.^30^

### Statistics

Statistical analysis was performed using Prism 10 Software (GraphPad Software, Inc). Statistical methods used were parametrical tests (t-test, one-way ANOVA, 2way ANOVA, mixed effects analysis) and the exact test per analysis is specified in the figure legends. Multiple testing was corrected for by Tukey's or Šídák's multiple comparison test. Assumptions for using parametrical tests were assessed visually by t-standardized residual plot (equal variance) or QQ plot (normality). If assumptions were not met, after careful evaluation, non-parametrical tests were used. Comparisons leading to p-values of ≤0.05 were considered statistically significant with p-values shown above the comparisons. p values > 0.05 are not shown and comparisons are considered non-significant. For TCR profiling with the R package Immunarch, the default test (Kruskal–Wallis test with Holm-Bonferroni correction) was performed. Correlations were determined by linear regression analysis. To test the effect of adoptive cell transfer into tumour-bearing HIS or NSG recipient mice on the outcome (tumour volume), a linear mixed effects model was fitted that considered the effect of the individual replicates (“experiment”) as a random effect (using R package lme4 and function lmer). Then, post hoc analysis was performed (using R package multcomp and function glht) by applying Tukey's multiple comparison test and multiple testing was corrected for with the Holm method. Baseline weight as a random effect was explored on the outcome (tumor volume) considering individual replicates (“experiment”) as a random factor by fitting a linear mixed model (R package lme4 and function lmer). We did not find an effect of baseline weight on tumor volume, so we did not include baseline weight as a random effect for the final analysis.

### Study approval

Animal experiments were conducted according to licenses approved by the veterinary office of the canton of Zurich, Switzerland (ZH049/20 and ZH067/2023).

### BioRender

Schematics were generated with a licensed version of BioRender.com.

### Role of funders

Funders did not have a role in study design, data collection, data analyses, interpretation or writing of report.

## Results

### Establishment of an autologous HIS mouse tumour model

**Fig. 1:**
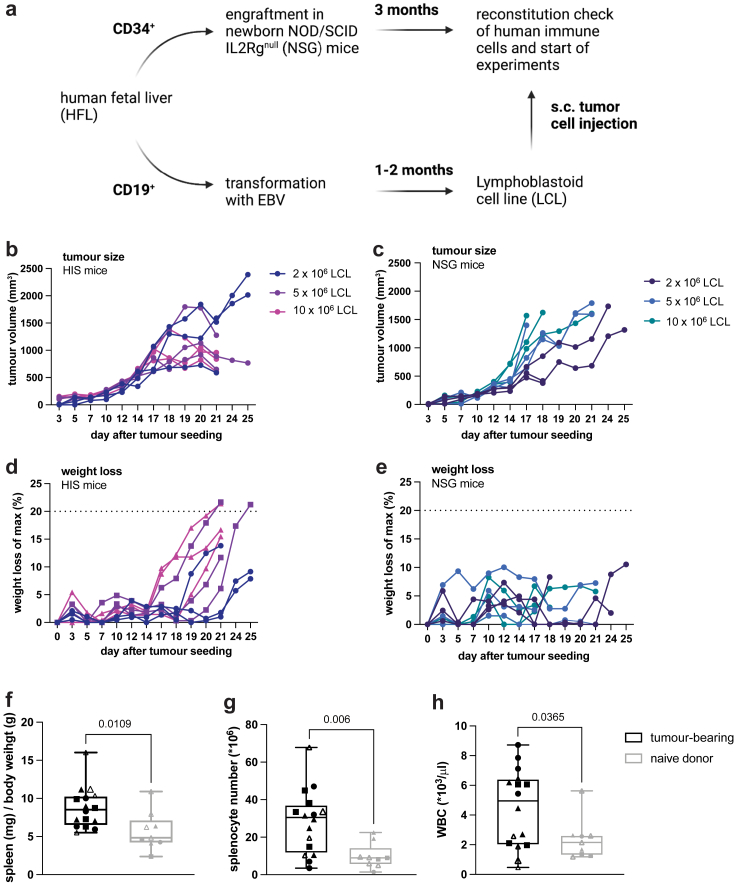
**LCL tumour growth kinetics and weight loss in autologous HIS mice and NSG mice**. LCL tumours were injected subcutaneously into the flank of mice and tumour size was assessed by calipering. **a**, schematic of generation of HIS mice and autologous tumour cells and subsequent injection in HIS mice. **b**, tumour volume in HIS mice after implantation of the indicated number of LCLs. **c**, Tumour volume in NSG mice after implantation of the indicated number of LCLs. (d, e) Relative weight loss calculated based on the maximum weight during the experiment after tumour cell implantation of the indicated number of LCLs in HIS (**d**) or NSG (**e**) mice. **f**, ratio of spleen weight (mg) to body weight (g), **g**, total splenocyte number and **h**, white blood cell counts in HIS mice injected with 5 × 10^6^ autologous LCL tumour cells or non-tumour bearing HIS mice (naïve) sacrificed 15–20 days after tumour implantation. b–e, n = 3 per tumour cell number, f-h, n(tumour-bearing) = 16, n(naïve) = 9 from 4 individual experiments, marked by individual symbols. Unpaired t-test, data shown as box and whiskers with the box from the 25 to 75 percentile and the median is shown as a line within the box; whiskers are shown from minimum to maximum data point.

We established and characterized a tumour model in which immunocompetent, humanized mice are subcutaneously implanted with autologous human tumour cells. For this aim, we isolated CD34^+^ hematopoietic progenitor cells (HPC) and CD19^+^ B cells from the same human HPC donor tissue. CD34^+^ HPCs were engrafted into NSG mice to reconstitute components of the human immune system (from here on referred to as human immune system (HIS) mice), while CD19^+^ B cells were transformed into lymphoblastoid cell lines (LCL) by infection with the oncogenic Epstein–Barr virus (EBV) (Fig. 1a).

To characterize LCL tumorigenicity and tumour growth in the presence or absence of an autologous human immune system, HIS mice reconstituted with HPCs autologous to the tumour or NSG mice were injected subcutaneously with LCLs. In both recipient strains, palpable tumours formed at the site of implantation. In HIS mice, in the presence of an endogenous immune system, tumours showed progressive growth until approximately day 18 and subsequently, a majority of tumours showed regression (Fig. 1b). In contrast, in NSG mice without an endogenous immune system, tumours increased in size until termination criteria were reached (Fig. 1c). These data suggested a functional antitumour response mediated by components of the reconstituted human immune system, i.e. immunocompetence, in our autologous HIS mouse tumour model.

### Endogenous antitumour immune response in HIS mice

To investigate the tumour regression that we observed in the presence of an autologous human immune system, we next characterized the immune response in tumour-bearing HIS mice. Around the same time of tumour regression, we found weight loss in HIS mice (Fig. 1d), but not in tumour-bearing NSG mice (Fig. 1e). This indicated immunopathology related to the endogenous human antitumour immune response, reminiscent of cancer cachexia^31^ rather than an effect of tumour growth per se. Additionally, we found increases in spleen-to-body weight ratio (p = 0.0109, unpaired t-test), total splenocyte count (p = 0.006, unpaired t-test) and white blood cell count in tumour-bearing HIS mice (p = 0.0365, unpaired t-test) (Fig. 1f–h), suggesting immune cell proliferation occurring in response to tumour growth. Baseline human immune cell reconstitution and T cell activation were comparable in tumour-bearing and naïve HIS mice (Supplemental Figure S1a). Furthermore, tumours in HIS mice showed infiltration by endogenous human immune cells, including CD8^+^ T cells (Supplemental Figure S1b–e).

**Fig. 2:**
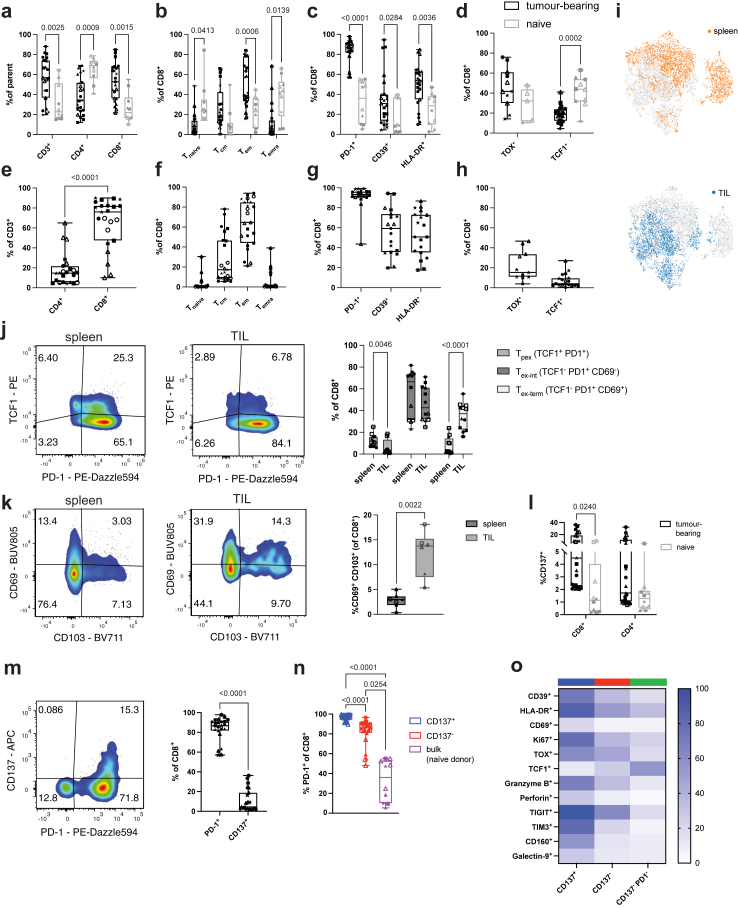
**T cell response in tumour-bearing HIS mice.** Splenocytes (a-d) and tumour-infiltrating lymphocytes (TIL) (e–h) were isolated from HIS mice 16–18 days after tumour implantation and analysed by flow cytometry. **a**, frequency of splenic T cells in tumour-bearing or naïve HIS mice; parent population refers to frequency (%) of CD3^+^ T cells within human CD45^+^ cells and CD4^+^ and CD8^+^ T cells within CD3^+^ T cells. **b**, CD8^+^ T cell differentiation defined as T_naïve_ (CD45RA^+^CD62L^+^), T_CM_ (CD45RA^−^CD62L^+^), T_EM_ (CD45RA^−^CD62L^−^), T_EMRA_ (CD45RA^+^CD62L^−^) in tumour-bearing or naïve HIS mice. **c and d**, expression of indicated markers on CD8^+^ T cells from spleen of tumour-bearing or naïve HIS mice. **e**, frequency of CD4^+^ and CD8^+^ T cells within TILs, gated on total CD3^+^ T cells. **f**, CD8^+^ T cell differentiation within TILs. **g and h**, expression of indicated markers on CD8^+^ T cells within TILs. **i**, UMAP of CD8^+^ T cells from spleen and TIL of tumour-bearing HIS mice, coloured for tissue of origin. **j**, expression of PD-1 and TCF1 in spleen and TIL of tumour-bearing HIS mice and quantification of CD8^+^ T_pex_ (TCF1^+^PD1^+^), T_ex-int_ (TCF1^−^PD1^+^CD69^−^), T_ex-term_ (TCF1^−^PD1^+^CD69^+^) in both tissues. **k**, expression of CD69 and CD103 in spleen and tumour of tumour-bearing HIS mice and quantification of T_RM_-like (CD69^+^CD103^+^) CD8^+^ T cells in both tissues. **l**, expression of CD137 on splenic CD8^+^ and CD4^+^ T cells of tumour-bearing or naïve HIS mice. **m**, expression and quantification of PD-1 and CD137 on splenic CD8^+^ T cells of tumour-bearing HIS mice. **n**, expression of PD-1 on splenic CD137^+^ or CD137^-^ CD8^+^ T cells in tumour-bearing HIS mice or bulk CD8^+^ T cells from naïve HIS mice. **o**, expression of individual markers on splenic CD8^+^ T cell populations from tumour-bearing HIS mice based on expression of CD137 and PD-1. a–d: n(tumour-bearing) = 11–23, n(naïve) = 5–10, 2way ANOVA with Šídák's multiple comparison test. Data are pooled from 2 to 5 independent experiments. e–f: n = 11–23, from 2 to 5 independent experiments. e, paired t-test, f-h, no statistical test performed. j, n = 12, from 3 independent experiments. 2way ANOVA with Šídák's multiple comparison test. k, n = 8, from 3 independent experiments. Paired t-test. l, n(tumour-bearing) = 23, n(naïve) = 10, from 6 independent experiments. 2way ANOVA with Šídák's multiple comparison test. m, n = 23, from 6 independent experiments. Paired t-test. n, n(tumour-bearing) = 11–23, n(naïve) = 5–10, from 6 independent experiments. Kruskal–Wallis test. o, n = 8–23, from 3 to 6 independent experiments. 2way ANOVA with Šídák's multiple comparison test. For each experiment, a different HPC donor was used for HIS mouse reconstitution and generation of autologous tumour. Data from individual experiments are indicated by different symbols, with individual mice from the same experiment indicated by the same symbol.

The frequency of human CD3^+^ T cells (Supplemental Figure S2) in tumour-bearing HIS mice in spleen (p = 0.0025, 2way ANOVA) (Fig. 2a) and circulation (p = 0.0315, 2way ANOVA) (Supplemental Figure S3a) increased significantly compared to naïve HIS mice. Moreover, tumour-bearing HIS mice showed an increased frequency of CD8^+^ T cells (p = 0.0015, 2way ANOVA), suggesting preferential expansion (Fig. 2a and Supplemental Figure S3a). These CD8^+^ T cells exhibited differentiation towards an effector memory (T_EM_) phenotype and, to a lower degree, central memory (T_CM_) phenotype, whereas non-tumour bearing HIS mice had higher frequencies of naïve T cells (T_naïve_) (Fig. 2b and Supplemental Figure S3b). Consistent with an activated and exhausted effector-like phenotype, CD8^+^ T cells from tumour-bearing HIS mice also showed enhanced expression of the exhaustion/activation markers PD-1 (p < 0.0001, 2way ANOVA), CD39 (p = 0.0284, 2way ANOVA) and HLA-DR (p = 0.0036, 2way ANOVA) (Fig. 2c and Supplemental Figure S3c) as well as trending towards increased TOX expression (Fig. 2d and Supplemental Figure S3d) while exhibiting significantly reduced levels of the progenitor/stemness-related marker TCF1 (p = 0.0002, 2way ANOVA)^32^ (Fig. 2d and Supplemental Figure S3d).

Similar to the increased CD8^+^ T cell frequencies in secondary lymphoid tissue (spleen) and circulation, tumours in HIS mice exhibited predominant infiltration by CD8^+^ T cells (Fig. 2e and Supplemental Figure S1d). The majority of autologous tumour-infiltrating human CD8^+^ T cells had a T_EM_-like phenotype, with a lower proportion of T_CM_ and greatly reduced proportions of T_EMRA_ and naïve T cells (Fig. 2f). Tumour-infiltrating CD8^+^ T cells displayed high expression of exhaustion and activation markers PD-1 (91.0% ± 12.1), CD39 (56.4% ± 22.8) and HLA-DR (53.9% ± 22.4) (Fig. 2g), low levels of TCF1 (5.6% ± 5.0) (Fig. 2h), while a fraction of tumour-infiltrating CD8^+^ T cells expressed TOX (21.7% ± 14.81) (Fig. 2h). Dimensionality reduction by UMAP revealed obvious differences of CD8^+^ T cell landscapes between TIL and spleen (Fig. 2i). Together, the phenotype of human CD8^+^ T cells in tumour-bearing HIS mice was reminiscent of CD8^+^ T cell phenotypes described in human cancer.^33^^,^^34^

Progenitor exhausted T cells (T_pex_; TCF1^+^PD-1^+^) play an important role for immunotherapy of human cancer. T_pex_ have been described as precursors of exhausted cytotoxic T cells (T_ex_; TCF1^−^PD-1^+^, further subdivided into TCF1^−^PD-1^+^CD69^−^ intermediate exhausted (T_ex-int_) and TCF1^−^PD-1^+^CD69^+^ terminally exhausted (T_ex-term_) T cells^35^), which exhibit effector function but reduced self-renewal and long-term survival, making T_pex_ cells an important reservoir of antitumour T cells.^35^, ^36^, ^37^ Interestingly, we detected an increased fraction of CD8^+^ T_pex_ cells in secondary lymphoid tissue (spleen) of tumour-bearing HIS mice compared with tumour (p = 0.0046, 2way ANOVA) (Fig. 2j). This distribution recapitulates human cancers, where a higher frequency of T_pex_ is found in lymph nodes compared to the tumour site.^36^ In addition, we found a significantly higher proportion of terminally exhausted CD8^+^ T cells (T_ex-term_; TCF1^−^PD-1^+^CD69^+^) within the tumour compared to the spleen, a secondary lymphoid organ (p < 0.0001, 2way ANOVA) (Fig. 2j), again consistent with what has been described in human cancer.^36^^,^^37^

Tissue-resident-memory (T_RM_) T cells (CD69^+^CD103^+^) within tumours are another T cell subset of interest for immunotherapy research. The presence of T_RM_ cells has been associated with a favourable outcome in certain cancer types and based on their expression of immune checkpoint molecules, tumour-reactive T_RM_ cells also constitute a target for immune checkpoint blockade.^38^ We detected T_RM_-like (CD69^+^CD103^+^) CD8^+^ T cells in tumour-bearing HIS mice and found a significantly higher abundance in tumour compared with spleen (p = 0.0022, paired t-test) (Fig. 2k).

The presence and identification of these human CD8^+^ T cell subsets, which are considered relevant in human cancer and are thought to play critical roles in response to immunotherapy, underscores the translational value of our humanized mouse model for immunotherapy research.

### Activated and exhausted-like, cytotoxic effector-like phenotype of human CD137^+^ CD8^+^ T cells

In human cancers, CD137 has been described as a marker for tumour-reactive T cells.^39^, ^40^, ^41^ We similarly identified a population of human CD137^+^ T cells in tumour-bearing mice (Fig. 2l) and characterized the CD8^+^ T cell population further. Likewise, PD-1 has been reported to be a marker of tumour-reactive T cells in human cancers, both in circulation and within TIL.^21^^,^^42^^,^^43^ We observed that of splenic CD8^+^ T cells from tumour-bearing HIS mice, only a small proportion (10.9% ± 11.2) were CD137 positive, whereas the majority of CD8^+^ T cells (83.5% ± 12.1) expressed PD-1 on their surface (Fig. 2m). Though there was a small but still significant difference (p < 0.0001, 2way ANOVA) between the overall high level of PD-1 expression on CD137^+^ CD8^+^ and CD137^−^ CD8^+^ T cells from tumour-bearing HIS mice, both these populations expressed PD-1 significantly more than bulk CD8^+^ T cells from naïve HIS mice (p < 0.0001 and p = 0.0254, respectively. 2way ANOVA) (Fig. 2n). These data suggested that CD137 alone might not be sufficient to delineate tumour-reactive T cell subsets. We therefore included a CD8^+^ T cell subset negative for both CD137 and PD-1 in our analysis to further explore and distinguish bona fide tumour-reactive from bystander CD8^+^ T cells in tumour-bearing HIS mice.

Indeed, compared to double negative CD137^−^PD-1^−^ CD8^+^ T cells, CD137^+^ CD8^+^ T cells and CD137^−^ CD8^+^ T cells displayed a T_EM_-like phenotype, while the double negative CD137^−^PD-1^−^ population was enriched in naïve-like T cells (Supplemental Figure S3e). Within the splenic CD137^+^ CD8^+^ T cell population, we found the highest expression of the exhaustion marker CD39, which has been described as a marker of CD8^+^ T cells with enhanced tumour reactivity across different human cancers,^1^^,^^44^, ^45^, ^46^, ^47^, ^48^ the activation marker HLA-DR as well as the activation and residency-related marker CD69,^49^ while this cell subset also had the strongest expression levels of Ki-67, indicating high proliferation (Fig. 2o and Supplemental Figure S3f). Additionally, within the CD137^+^ CD8^+^ T cell population, we observed significantly increased expression of TOX, a transcription factor associated with exhaustion^50^ and drastically reduced levels of the progenitor/stemness-related transcriptional regulator TCF1^32^ (Fig. 2o and Supplemental Figure S3g). Several makers related to T cell exhaustion and activation^51^^,^^52^ (TIGIT, TIM3, CD160, Galectin-9) were similarly upregulated on CD137^+^ CD8^+^ T cells (Fig. 2o and Supplemental Figure S3h). Finally, CD137^+^ CD8^+^ T cells displayed the highest levels of the cytolytic molecules granzyme B and perforin compared with CD137^−^ CD8^+^ T cells or CD137^−^PD-1^−^ CD8^+^ T cells from tumour-bearing HIS mice (Fig. 2o and Supplemental Figure S3i).

The CD137^+^ CD8^+^ T cell population contained a significantly higher proportion of T_ex-term_ cells in both spleen and tumour and a significantly lower frequency of T_pex_ in the tumour relative to the CD137^−^ CD8^+^ T cell population (Supplemental Figure S3j and 3k). As CD69 is expressed on subsets of both exhausted as well as T_RM_-like T cells,^35^ we quantified CD8^+^ T_RM_-like (CD69^+^CD103^+^) T cells and found no difference between CD137^+^ and CD137^−^ T cells in the spleen (Supplemental Figure S3l). In the tumour, where an overall higher proportion of T_RM_-like T cells was present compared to spleen (p = 0.0022, paired t-test) (Fig. 2k), the CD137^+^ CD8^+^ T cell population contained significantly fewer T_RM_-like T cells compared to CD137^−^ CD8^+^ T cells (p = 0.0039, 2way ANOVA) (Supplemental Figure S3m). Thus, the majority of CD137^+^ CD8^+^ T cells did not exhibit a T_RM_-like phenotype but consisted of more T_ex-term_ cells relative to CD137^−^ CD8^+^ T cells with only a minor proportion of T_pex_ cells.

Analysis of CD8^+^ T cell clusters (Supplemental Figure S4a) from spleen and tumour demonstrated that CD137 expressing clusters 2, 7 and 10 (Supplemental Figure S4b and c) co-expressed a large number of markers related to activation/exhaustion (TIGIT, TIM3, PD-1, CD39, CD160). CD8^+^ T cells in cluster 7, the majority of which was localized in the tumour (Supplemental Figure S4b), additionally expressed LAG-3 (Supplemental Figure S4c). Moreover, co-expression analysis confirmed the presence of a T_pex_-like cluster (TCF1^+^PD1^+^CD69^−^), which was preferentially found in the spleen (cluster 3). Also, a T_RM_-like cluster (CD69^+^CD103^+^) was detected, which was preferentially found in tumour (cluster 5) (Supplemental Figure S4c).

Altogether, de novo arising highly proliferative CD137^+^ CD8^+^ T cells exhibited an activated and exhausted-like, cytotoxic effector and least stem-like phenotype relative to the other human CD8^+^ T cell populations we identified in tumour-bearing HIS mice.

### Maintained expansion capacity and enrichment of *in vitro* tumour reactivity within an activated and exhausted-like human CD8^+^ T cell subset

**Fig. 3:**
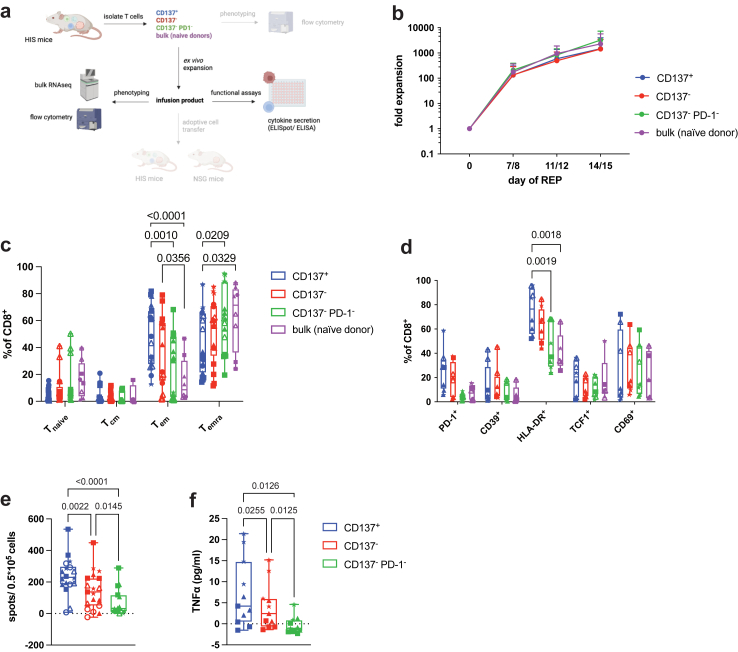
**Activated and exhausted-like human CD8**^**+**^**T cells derived from tumour-bearing HIS mice can be expanded *ex vivo* and exhibit superior tumour-specific cytokine production. a**, schematic of generation, expansion and characterization of T cells from HIS mice bearing autologous LCL tumours. **b**, fold expansion of FACS sorted splenic CD8^+^ T cells from tumour-bearing HIS mice (CD137^+^, CD137^-^ and CD137^−^PD1^-^) or naïve HIS mice (bulk). **c**, CD8^+^ T cell differentiation after *ex vivo* expansion defined as T_naïve_ (CD45RA^+^CD62L^+^), T_CM_ (CD45RA^−^CD62L^+^), T_EM_ (CD45RA^−^CD62L^−^), T_EMRA_ (CD45RA^+^CD62L^−^). **d**, expression of indicated markers after *ex vivo* expansion. **e**, IFN-γ ELISpot of expanded T cells in 5:1 (E:T) co-culture with autologous LCL tumour cells for 24 h. Spot count is normalized to the spots produced by expanded CD8^+^ bulk T cells from naïve HIS mice. **f**, TNF⍺ ELISA of supernatant of expanded T cells in co-culture (5:1, E:T) with autologous LCL tumour cells for 24 h. TNF⍺ concentration is normalized to the TNF⍺ secretion from expanded CD8^+^ bulk T cells derived from naïve mice. b, n = 8–19, from 5 to 6 independent experiments. Matched 2way ANOVA with Tukey's multiple comparison test. c, n = 8–19, from 5 independent experiments. 2way ANOVA with Tukey's multiple comparison test. d, n = 5–8, from 4 independent experiments. 2way ANOVA with Tukey's multiple comparison test. e, n = 16–19, from 5 independent experiments. Mixed-effects analysis with matching and Tukey's multiple comparison test. f, n = 11, from 3 independent experiments. Repeated measures one-way ANOVA with Tukey's multiple comparison test. For each experiment, a different HPC donor was used for HIS mouse reconstitution and generation of autologous tumour. Data from individual experiments are indicated by different symbols, with individual mice from the same experiment indicated by the same symbol.

To investigate tumour reactivity of CD137^+^ CD8^+^ T cells relative to the other T cell subsets, splenic T cells were expanded *ex vivo* (Fig. 3a). Using a rapid expansion protocol,^21^ we observed an average expansion of >1000-fold after 14 days for all subsets. Interestingly, although terminally exhausted CD8^+^ T cells have generally been thought to contain reduced proliferative potential,^53^ there were no significant differences in the expansion capacity of CD137^+^ CD8^+^ T cells displaying the most exhausted-like, T_ex-term_ enriched phenotype (Fig. 2O and Supplemental Figure S3j and k) compared to the other CD8^+^ T cell populations from tumour-bearing HIS mice or bulk CD8^+^ T cells from naïve HIS mice (Fig. 3b). All of the *ex vivo* expanded T cell subsets from tumour-bearing HIS mice exhibited differentiation biased to T_EM_ and T_EMRA_-like phenotypes (Fig. 3c). While prior to *ex-vivo* expansion both PD-1 and CD39 were strongly increased in CD137^+^ CD8^+^ T cells (Fig. 2, o and n), both markers were no longer significantly upregulated after expansion. In contrast, HLA-DR remained upregulated on CD137^+^ CD8^+^ T cells relative to CD137^−^PD1^−^ CD8^+^ (p = 0.0019, 2way ANOVA) and bulk CD8^+^ T cells from naïve mice (p = 0.0018, 2way ANOVA) (Fig. 3d).

After co-culture with autologous tumour cells, expanded CD137^+^ CD8^+^ T cells showed the highest IFN-γ production by ELISpot, which gradually decreased from CD137^−^ CD8^+^ T cells to CD137^−^PD-1^−^ CD8^+^ T cells (Fig. 3e). Similarly, TNFα secretion was highest in supernatants containing CD137^+^ CD8^+^ T cells after co-culture with autologous tumour cells and lowest in CD137^−^PD-1^−^ CD8^+^ T cells (Fig. 3f).

Together, these data indicated enrichment of tumour recognition within CD137^+^ CD8^+^ T cells that maintained *ex vivo* proliferative potential, while double negative CD137^−^PD-1^−^ CD8^+^ T cells behaved as bystander cells.

### Transcriptome analysis reveals tumour-reactive signature in CD137^+^ CD8^+^ T cells

**Fig. 4:**
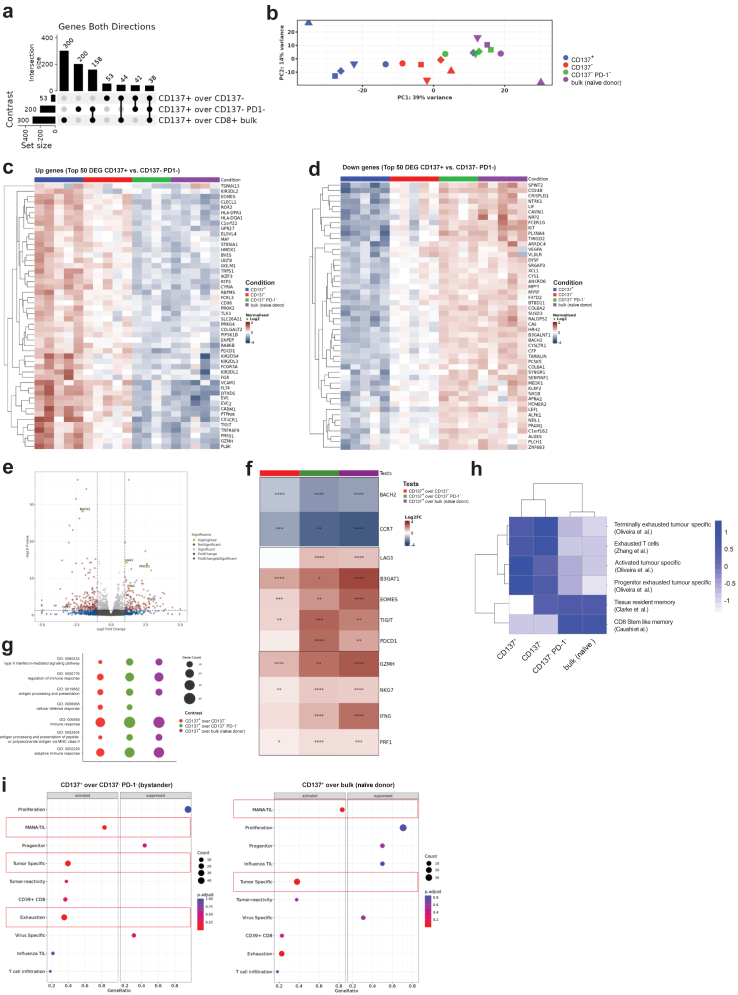
**Transcriptomic profiling of tumour-reactive CD137**^**+**^**CD8**^**+**^**T cells**. Transcriptome analysis and pathway analysis of expanded CD8^+^ T cells from tumour-bearing HIS mice (CD137^+^, CD137^−^ and CD137^−^PD1^−^) or naïve HIS mice (bulk). **a**, Upset plot (intersect) showing number of differentially expressed genes between groups in bulk RNAseq. p(FDR) < 0.05, log2 FC > 1.5. **b**, PCA plot of RNAseq showing PC1 and PC2. **c**, Top 50 upregulated and **d**, downregulated genes of T cell subsets based on the DEG between CD137^+^ versus CD137^−^PD1^−^ CD8^+^ T cells. p(FDR) < 0.05, log2 FC > 1.5. **e**, Volcano plot showing DEG between CD137^+^ and CD137^−^PD1^−^ CD8^+^ T cells with genes of interest highlighted in yellow. **f**, differential expression of genes of interest between groups. **g**, Overrepresentation analysis (ORA) of upregulated pathways in CD137^+^ CD8^+^ T cells. **h**, Gene module score analysis of published signatures described on the right. **i**, Gene set enrichment analysis (GSEA) of signatures described on the y-axis. Gene ratio (# genes related to GO term/total number of sig genes) is displayed on the x-axis. Signatures with an adjusted p-value <0.05 are highlighted with a red box. Shown are data from 4 to 5 individual experiments, each experiment with different human HPC donor for reconstitution of HIS mice and autologous tumour.

To gain insight into differentially expressed genes (DEG) in tumour-reactive CD137^+^ CD8^+^ T cells versus CD137^-^ CD8^+^ T cells and bystander CD137^−^PD-1^−^ CD8^+^ T cells as well as bulk CD8^+^ T cells from naïve HIS mice, whole transcriptome RNA sequencing of *ex vivo* expanded T cell subsets was performed. We found a higher number of DEG for the tumour-reactive (CD137^+^) gene set versus bystander CD8^+^ T cells from tumour-bearing HIS mice (200 DEG) and versus bulk CD8^+^ T cells from naïve HIS mice (300 DEG) in comparison to the number of DEG we found for the comparison against CD137^−^ CD8^+^ T cells (53 DEG) (Fig. 4a), the subset with the second highest tumour reactivity *in vitro* (Fig. 3e and f). Principal component analysis (PCA) was consistent with functional data of tumour-specific cytokine production (Fig. 3e and f), showing that the 4 subsets clustered in a gradual way from tumour-reactive (CD137^+^) to less tumour-reactive (CD137^−^) and bystander (CD137^−^PD-1^−^) CD8^+^ T cells and finally to bulk CD8^+^ T cells from naïve HIS mice, with the biggest overlap between CD137^−^PD-1^−^ bystander CD8^+^ T cells and bulk human CD8^+^ T cells from naïve HIS mice (Fig. 4b).

The transcriptomic profile that we found in tumour-reactive CD137^+^ CD8^+^ T cells suggested an exhausted/activated-like phenotype with increased expression of *TNFRSF9*, *LAG3*, *PDCD1, TIGIT* and *HLA-DPA1/HLA-DQA1* (Fig. 4c,e and f). We also observed enhanced expression of effector molecules associated with cytotoxicity (*GZMH, NKG7* and *PRF1*) as well as *IFNG* (Fig. 4c,e and f). Expression of the naïve or stem-like genes *CCR7*, *LEF1* and *BACH2* were diminished (Fig. 4d–f) while the senescence marker CD57^54^ (*B3GAT1*) and the transcription factor *EOMES* showed the highest transcript level in the CD137^+^ subset (Fig. 4c and f). Although EOMES has been shown to be involved in T cell exhaustion, it has also been reported to be commonly co-expressed with effector molecules in effector T cells and to be essential for anticancer T cell function.^55^^,^^56^

Among upregulated Gene Ontology (GO) terms comparing tumour-reactive CD137^+^ CD8^+^ T cells against the other three populations, we found the terms interferon-gamma mediated signalling (GO:0060333), antigen processing and presentation (GO:0019882), antigen processing and presentation of peptide or polysaccharide antigen via MHC class II (GO:0002504), immune response (GO:0006955) and adaptive immune response (GO:0002250) (Fig. 4g). Upregulation of these pathways in the CD137^+^ population indicated stronger T cell activation and response in comparison to the other populations.

To determine T cell states after *ex vivo* expansion, we compared our transcriptome data to published datasets of different T cell states from human cancers.^33^^,^^48^^,^^57^, ^58^, ^59^, ^60^, ^61^ A high overlap of signatures originally found in tumour-specific CD8^+^ T cells from human cancers describing terminally exhausted, exhausted, activated, and progenitor exhausted T cell states was found for CD137^+^ CD8^+^ T cells (Fig. 4h). Overlap with these different signatures might hint to a degree of heterogeneity within the CD137^+^ CD8^+^ T cell population after *ex vivo* expansion but confirmed the overall activated/exhausted-like state of these cells that we already observed before *ex vivo* expansion. Signatures for tissue-resident memory and CD8^+^ stem-like memory were downregulated in the CD137^+^ CD8^+^ population. Strikingly, comparing tumour-reactive CD137^+^ CD8^+^ T cells to bystander CD137^−^PD-1^−^ CD8^+^ T cells or bulk CD8^+^ T cells from naïve HIS mice, gene sets that have been described to define tumour-reactive T cells in patients with cancer were significantly upregulated (‘MANA TIL’,^60^ ‘tumour specific’^58^) (Fig. 4i). These results suggested that the antitumour T cell response in autologous tumour-bearing HIS mice mirrors relevant aspects of the T cell response in patients with cancer, thus further supporting the potential translational value of our model.

### Correlation of clonal expansion with effector function

**Fig. 5:**
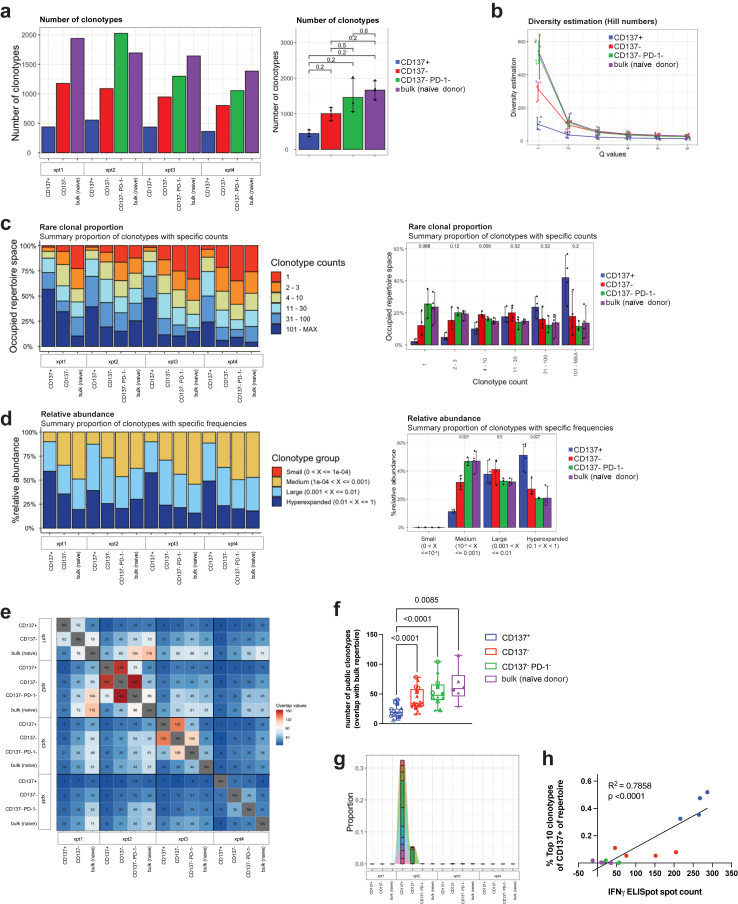
**TCR profiling of tumour-reactive CD137**^**+**^**CD8**^**+**^**T cells. a**, total number of individual clonotypes found per population. Left: data from individual experiments, right: pooled data for group analysis. Kruskal–Wallis test with Holm-Bonferroni correction. **b**, TCR (CDR3 of *TRA*, *TRB*, *TRG* and *TRD*) sequence sample diversity estimation using Hill numbers method, with Q = 1 describing the Shannon diversity. **c**, rare clonal proportion showing the occupied repertoire space by clonotypes with defined counts (1, 2–3, 4–10, etc.). Left: data from individual experiments, right: pooled data for group analysis. Kruskal–Wallis test with Holm-Bonferroni correction. **d**, relative abundance of clonotypes with defined frequencies (size). Left: data from individual experiments, right: pooled data for group analysis. Kruskal–Wallis test with Holm-Bonferroni correction. **e**, repertoire overlap analysis cross-comparing every population from every experiment (each with different donor). **f**, repertoire overlap comparing the repertoire of bulk CD8^+^ T cells from naïve HIS mice to the populations from tumour-bearing HIS mice from individual experiments (each with different donor; data from individual experiments are indicated by different symbols). Mixed-effects analysis with Tukey's multiple comparisons test. **g**, tracking of clonotypes over populations. The top 10 most abundant clonotypes of the TCR repertoire of CD137^+^ CD8^+^ T cells from one representative experiment are shown. **h**, proportion of the top 10 most abundant clonotypes (from repertoires of CD137^+^ CD8^+^ T cells) in the repertoire of all populations, correlated with the spot count of IFN-γ ELISpot. Shown are data from 4 individual experiments, each experiment with different human HPC donor for reconstitution of HIS mice and autologous tumour. Correlation was determined by linear regression analysis.

As another surrogate for tumour recognition, we analysed clonality of the CD8^+^ T cell receptor (TCR) repertoire, anticipating tumour-reactive CD8^+^ T cells would have higher clonal expansion and thus lower diversity than bystander CD8^+^ T cells. Indeed, we found the total number of clonotypes tending to be the smallest in the tumour-reactive CD137^+^ subset, and to gradually increase from the less tumour-reactive CD137^−^ subset to CD137^−^PD-1^−^ bystander CD8^+^ T cells to bulk CD8^+^ T cells from naïve HIS mice (Fig. 5a), following the gradient of tumour recognition we observed by tumour-specific cytokine production *in vitro* (Fig. 3d and e). Likewise, the estimated sample diversity for the tumour-reactive CD137^+^ subset was lower than for the other populations (Fig. 5b and Supplemental Figure S5a). In line with this, the CD137^+^ population had a reduced abundance of rare or very small clonotypes (Fig. 5c and Supplemental Figur S5b and c). Conversely, tumour-reactive CD137^+^ CD8^+^ T cells from tumour-bearing HIS mice demonstrated a significantly higher abundance of hyperexpanded clones (Fig. 5d) with the 20 biggest clones occupying a significantly larger repertoire space than in the other populations, suggesting polyclonal expansion (Supplemental Figure S5b).

To track the overlap of clonotypes between individual experiments (i.e. donor dependency) and populations, we next performed TCR repertoire overlap analysis (Fig. 5e). We found that the tumour-reactive CD137^+^ population showed the biggest overlap with the CD137^-^ subset and only to a minor extend an overlap with CD137^−^PD-1^−^ bystander CD8^+^ T cells or bulk CD8^+^ T cells from naïve HIS mice (Fig. 5e and g). Also, analysing the repertoire of the bulk naïve CD8^+^ T cell population for each experiment by cross-comparing to each population from each experiment, the overlap with the CD137^+^ population was significantly less than the overlap with every other population (Fig. 5f). These findings suggested that clonotypes arising due to the conditions of expansion were less present in the CD137^+^ population and that other, presumably tumour-reactive, clonotypes expanded.

In addition, we observed that the expansion of individual bona fide tumour-reactive clonotypes was highly donor specific. When comparing the 10 most abundant clonotypes of the CD137^+^ population per experiment (i.e. per donor), almost no overlap with the CD137^+^ TCR repertoire of any other experiment was detected (Fig. 5g and Supplemental Figure S5d). Finally, to explore an association of clonal expansion with functionality, we correlated the abundance of the top 10 bona fide tumour-reactive clonotypes of the CD137^+^ population with *in vitro* tumour reactivity and found a highly significant correlation of clone size abundance and tumour-specific IFN-γ production by CD8^+^ T cells (Fig. 5h). Overall, these data indicated that CD137^+^ CD8^+^ T cells derived from tumour-bearing HIS mice expanded polyclonally in a human donor- and tumour-specific manner.

### Human CD8^+^ T cells with an activated and exhausted-like phenotype reduce tumour growth *in vivo*

**Fig. 6:**
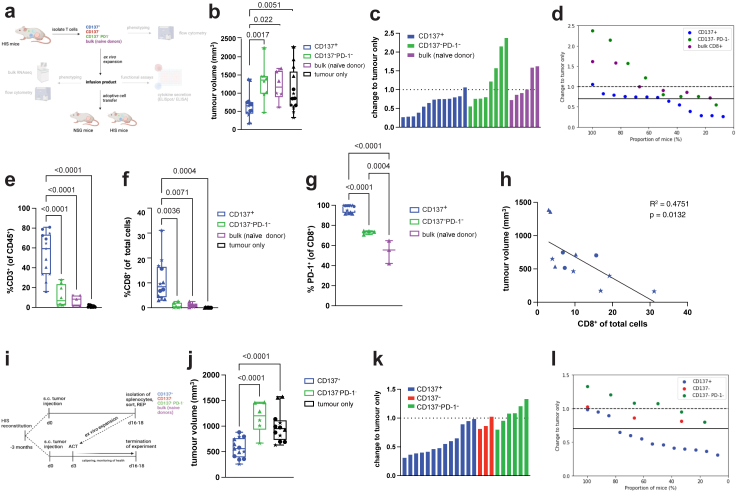
**CD137**^**+**^**CD8**^**+**^**T cells from tumour-bearing HIS mice show superior anticancer activity in NSG and HIS recipients bearing autologous tumours. a**, schematic of generation of tumour-reactive T cells and subsequent ACT. NSG mice were injected with 2 × 10^6^ LCL s.c. in the flank and after three days, 10 × 10^6^*ex vivo* expanded T cells were adoptively transferred intravenously. Transferred T cells and LCL tumours were autologous to each other. **b**, tumour volume on the day of sacrifice in NSG recipient mice after ACT of the indicated cell populations. **c**, waterfall plot of tumour size in NSG recipient mice of ACT on the day of sacrifice relative to the tumour volume of control mice (no ACT). Bars depict individual mice. **d**, proportion of NSG recipient mice showing partial response (≥30% tumour reduction compared to control NSG mice) after ACT of the indicated cell population. **e**, frequency of CD3^+^ T cells (% of human CD45^+^ cells) in TIL of NSG mice after ACT of the indicated cell populations, measured by flow cytometry. **f**, frequency of CD8^+^ T cells (% of total cells) in tumours of NSG mice after ACT of the indicated cell populations, measured by immunohistochemistry (IHC). **g**, frequency of PD-1 expression on CD8^+^ T cells in TIL of NSG mice after ACT of the indicated cell populations, measured by flow cytometry. **h**, correlation between tumour volume and infiltration of CD8^+^ T cells (measured by IHC) in tumours of NSG mice after adoptive transfer of CD137^+^ CD8^+^ T cells. **i**, schematic of ACT. CD137^+^ CD8^+^ T cells, CD137^-^ CD8^+^ T cells and CD137^−^PD-1^−^ CD8^+^ T cells were isolated from spleen of tumour-bearing HIS mice or bulk CD8^+^ T cells from spleen of naïve HIS mice and expanded *ex vivo*. Recipient HIS mice were injected with 2 × 10^6^ LCL s.c. in the flank and after three days, *ex vivo* expanded T cells were adoptively transferred intravenously. Tumour-bearing HIS recipient mice received 2 × 10^6^ T cells without prior conditioning/lymphodepletion. Donor and recipient HIS mice as well as LCL were autologous to each other. **j**, tumour volume on the day of sacrifice in HIS recipient mice after ACT of the indicated cell populations. **k**, waterfall plot of tumour size on the day of sacrifice of HIS mice receiving ACT relative to the tumour volume of control HIS mice (no ACT). Bars depict individual mice. **l**, proportion of HIS recipient mice showing partial response (≥30% tumour reduction compared to control HIS mice) after ACT of the indicated cell population. b–d, n(CD137^+^; tumour only) = 13, from 3 independent experiments. n(CD137^−^PD-1^−^) = 8, from 2 independent experiments. n(bulk) = 6, from 2 independent experiments. b, Linear mixed model with Tukey's multiple comparison test and Holm correction. e, f, n = 5–13, from 2 to 3 independent experiments. One-way ANOVA with Tukey's multiple comparison test. g, n = 3–12, from 1 to 3 independent experiments. One-way ANOVA with Tukey's multiple comparison test. Only data points in which >100 CD8^+^ events were recorded are shown. h, n(CD137^+^) = 12, from 3 independent experiments. Correlation was determined by linear regression analysis. j, n(CD137^+^) = 13, n(CD137^−^PD-1^−^) = 6, from 2 independent experiments, n(tumour only) = 11, from 3 independent experiments. Linear mixed model with Tukey's multiple comparison test and Holm correction. For each experiment, a different HPC donor was used for HIS mouse reconstitution and generation of autologous tumour. Data from individual experiments are indicated by different symbols, with individual mice from the same experiment indicated by the same symbol.

We next performed adoptive cell transfer (ACT) experiments of *ex vivo* expanded CD8^+^ T cells in NSG mice previously engrafted with autologous tumours (Fig. 6a). We initially selected NSG mice as recipients to unambiguously track the transferred T cells and to be able to distinguish their anticancer activity from the endogenous antitumour T cell response in HIS mice. In NSG mice, only ACT with expanded CD137^+^ CD8^+^ T cells resulted in reduced tumour growth in a substantial number of recipients, while transfer of bystander CD8^+^ T cells from tumour-bearing HIS mice or bulk CD8^+^ T cells from naïve HIS mice did not (Fig. 6b–d). Specifically, adoptive transfer of human CD137^+^ CD8^+^ T cells from tumour-bearing HIS donors resulted in a partial response (≥30% tumour reduction compared to ‘tumour only’ group) in almost half of recipients (6 out of 13 tumour-bearing NSG recipients, 46%), while this was observed in only 13% of NSG mice treated with CD137^−^PD-1^−^ CD8^+^ T cells from tumour-bearing HIS mice (1/8) and no such response (0%) was seen in NSG recipients of bulk CD8^+^ T cells from naïve HIS mice (0/6) (Fig. 6d).

Furthermore, a higher frequency of T cells was found in tumour (Fig. 6e and f), blood (Supplemental Figure S6a) and spleen (Supplemental Figure S6b) of tumour-bearing NSG mice receiving CD137^+^ CD8^+^ T cells compared to recipients transferred with any of the other cell populations. Flow cytometric analysis of TIL revealed high CD3^+^ T cell frequencies (53.65% ± 21.94) after ACT of expanded CD137^+^ CD8^+^ T cells, but only 11.64% ± 11.0 after transfer of bystander CD8^+^ T cells and 7.25% ± 5.38 after transfer of bulk CD8^+^ T cells from naïve HIS mice (Fig. 6e). Likewise, immunohistochemistry of tumours of NSG mice confirmed T cell infiltration selectively after ACT with CD137^+^ CD8^+^ T cells (Fig. 6f).

Interestingly, while infusion products mainly contained a mix of T_EM_-like and T_EMRA_ CD8^+^ T cells (Fig. 3c), almost exclusively T_EM_-like cells were recovered from blood (Supplemental Figure S6c), spleen (Supplemental Figure S6d) and TIL (Supplemental Figure S6e) of recipient NSG mice. Of note, whereas PD-1 was highly expressed on splenic CD137^+^ CD8^+^ T cells in tumour-bearing HIS mice (96.49% ± 3.1) (Fig. 3c), PD-1 expression on this population decreased during antigen-independent *ex vivo* expansion (32.45% ± 29.17) (Supplemental Figure S6f). However, after adoptive transfer of expanded CD137^+^ CD8^+^ T cells into tumour-bearing NSG mice, PD-1 expression among CD8^+^ T cells increased again and was significantly higher in TIL (96.7% ± 3.65) (Fig. 6g), blood (Supplemental Figure S6g) and spleen (Supplemental Figure S6h) compared to NSG mice transferred with bystander CD8^+^ T cells (p < 0.0001, one-way ANOVA) or bulk CD8^+^ T cells (p < 0.0001, one-way ANOVA). Apart from the superior tumour control, these data further indicated enrichment of tumour reactivity within CD137^+^ CD8^+^ T cells, with very high PD-1 expression serving as a potential marker for productive tumour recognition, but not loss of effector function. As another index of the anticancer activity of CD137^+^ CD8^+^ T cells, the degree of tumour infiltration with CD8^+^ T cells after ACT was inversely correlated with tumour size (p = 0.0132, linear regression) (Fig. 6h). Thus, the higher frequency of CD8^+^ T cells in tumours was likely driven by increased tumour recognition of transferred CD137^+^ CD8^+^ T cells, and these cells mediated tumour cell killing.

Lastly, ACT of tumour-reactive T cells into autologous tumour-bearing HIS recipient mice (Fig. 6i) presented an opportunity to model polyclonal TIL transfer therapy in patients with cancer^62^ in an immunocompetent humanized tumour setting. As observed with NSG recipients, transfer of polyclonal tumour-reactive CD137^+^ CD8^+^ T cells into autologous tumour-bearing HIS recipient mice reduced tumour growth (Fig. 6j–l). A partial response (≥30% tumour reduction compared to ‘tumour only’ group) was observed in 77% of HIS mice (10/13) receiving adoptive transfer of autologous human CD137^+^ CD8^+^ T cell derived from tumour-bearing HIS donors, while no such response (0%) was observed in any of the HIS mice (0/9) adoptively transferred with autologous CD137^-^ or CD137^−^PD-1^−^ CD8^+^ T cells derived from tumour-bearing HIS donors (Fig. 6l). Baseline HIS reconstitution and T cell activation before tumour injection and adoptive cell transfer did not differ between recipient groups (Supplemental Figure S6i). Performing post-hoc sex-based analysis, we did not observe differences between male and female HIS (Supplemental Figure S7a) or NSG (Supplemental Figure S7b) recipient mice within the same treatment group. Importantly, efficacy of adoptive transfer was achieved without prior conditioning of HIS recipient mice or cytokine support, and the number of transferred tumour-reactive CD137^+^ CD8^+^ T cells was fivefold lower compared with transfer in NSG recipients. These data established superior anticancer activity of CD137^+^ CD8^+^ T cells with an activated and exhausted-like phenotype in the most complete humanized setting.

Altogether, these adoptive transfer experiments confirmed anticancer activity of human CD137^+^ CD8^+^ T cells that developed in autologous tumour-bearing HIS mice, suggesting that our model captured relevant aspects of the immunobiology of human antitumour T cell responses.

## Discussion

Using in-depth phenotypic analyses, we established the presence of major, widely recognized human CD8^+^ T cell subsets known to be important in tumour immunology, like progenitor exhausted (T_pex_; TCF1^+^PD-1^+^), terminally exhausted (T_ex-term_; TCF1^−^PD-1^+^CD69^+^) or tissue resident-like (T_RM_; CD69^+^CD103^+^) T cells in our autologous humanized tumour model. Interestingly, we found CD8^+^ T_pex_ cells to be more abundant in secondary lymphoid tissue (spleen) compared to tumour and vice versa CD8^+^ T_ex-term_ cells to be more abundant in tumour compared to spleen. This seems consistent with the mechanisms of formation of antitumour immunity as currently understood.^63^ Likewise, tissue-resident-like (T_RM_) T cells were enriched in the tumour relative to spleen, as would be expected. Furthermore, we identified a proliferative human CD8^+^ T cell subset in tumour-bearing HIS mice that expressed activation and exhaustion markers including very high levels of PD-1 and CD39 while having low expression of the progenitor marker TCF1 but high expression of TOX. This CD137^+^ CD8^+^ T cell subset, while being PD-1^hi^CD39^+^, displayed increased abundance of a T_ex-term_ phenotype with co-expression of inhibitory receptors like TIM-3, TIGIT, CD160 and galectin-9. At the same time, CD137^+^ CD8^+^ T cell contained fewer T_RM_-like cells, which was reflected by a relative lack of a tissue-resident signature even after expansion. However, we note that the expansion, which was necessary to achieve the cell numbers required for adoptive transfer, may have led to some alteration of populations, as evidenced, for example, in the downregulation of PD-1 and CD39 on the protein level after expansion. Nevertheless, most activation and exhaustion markers were still increased on the transcript level in expanded CD137^+^ CD8^+^ T cells relative to the other populations. Furthermore, expanded CD137^+^ CD8^+^ T cells from tumour-bearing HIS mice showed enrichment in gene signatures of exhaustion. Functionally, CD137^+^ CD8^+^ T cells exhibited increased tumour reactivity as demonstrated by enhanced cytokine production after coculture with autologous tumour cells. Importantly, by means of adoptive transfer, we provide direct *in vivo* evidence of superior anticancer activity of these human tumour-reactive effector CD8^+^ T cells displaying an activated and exhausted-like phenotypic state. Thus, in this study, a reservoir of tumour-reactive CD8^+^ T cells was identified based on the expression of CD137, and we observed superior anticancer activity of the CD137^+^ subset compared to the CD137^-^ groups. However, we did not investigate whether CD137 is superior to other established markers for tumour-reactive T cells, such as CD39 or PD-1 and further research is required to determine which marker (or combination of markers) proves most useful in identifying tumour-reactive T cells.

We have leveraged this human CD137^+^ CD8^+^ T cell subset arising de novo in tumour-bearing HIS mice for transcriptomic analysis and TCR profiling to demonstrate across donors overlap with tumour-reactive CD8^+^ T cell signatures from human cancers^58^^,^^60^ as well as tumour-induced polyclonal expansion. Polyclonality of neoantigen-specific CD8^+^ T cells in blood and tumour has recently been shown to be associated with response to PD-1 immunotherapy in patients with melanoma.^16^ Congruence of our model data across donors with these clinical benchmarks suggests robustness and translational validity of our findings.

Of note, CD39^+^CD8^+^ T cells expressing high levels of PD-1 have been described to be enriched for tumour reactivity and clonal proliferation, predicting efficacy of immunotherapy in human lung cancer.^48^ Likewise, the abundance of intratumoral CD39^+^CD8^+^ T cells displaying upregulated exhaustion markers has been shown to be prognostic for improved progression-free and overall survival in individuals with treatment-naïve early stage lung cancer.^64^ Furthermore, PD-1^hi^CD39^+^ CD8^+^ T cells correlated with improved survival in patients with endometrial cancer^47^ and the signature of similar CD8^+^ T cells with increased TOX expression predicted survival in patients with breast cancer.^46^ Recently, pre-existing exhausted-like tumour-reactive CD8^+^ T cells have been associated with favourable outcome to immunotherapy in head and neck cancer.^65^ On the other hand, higher abundance of TCF1^+^ stem-like CD8^+^ T cells with a CD39^−^CD69^−^ phenotype in TIL products was associated with better response after TIL transfer in patients with melanoma.^66^ However, by single-cell analysis of lung tumours, this phenotype could not be identified in neoantigen-reactive T cells.^67^ While we have not addressed and thus cannot exclude a contribution of the minor TCF1^+^ fraction within the human tumour-reactive CD8^+^ T cells in our model, our data emphasize the key importance of activated and exhausted-like effector CD8^+^ T cells in the antitumour immune response. The observation that activated and exhausted-like human CD8^+^ T cells exhibit enhanced functionality compared to CD8^+^ T cells lacking inhibitory receptors has also been reported for virus specific T cells in SARS-CoV-2 infection,^68^ reinforcing the notion beyond the context of cancer that inhibitory receptors do not necessarily identify dysfunctional T cells.

The striking phenotypic similarity of tumour-reactive CD8^+^ T cells that have been shown to correlate with clinical benefit and the polyclonally expanded human tumour-reactive CD8^+^ T cells from tumour-bearing HIS mice that we demonstrated to possess superior *in vivo* anticancer activity highlights the translational potential of our model. Hence, the immunocompetent human tumour model that we developed using HIS mice with human immune cells reconstituted from hematopoietic progenitor cells that are autologous to tumour might bridge a gap between syngeneic mouse tumour models and human-specific antitumour immune responses.^13^^,^^69^ Our model demonstrates hallmarks of human antitumour T cell responses and thus may provide a tool that should allow further investigation of drivers of anticancer activity in human CD8^+^ T cells in cancer immunotherapy research. Overall, the endogenous human immune system in HIS mice markedly influenced tumour and host, allowing for experimental interrogation of the interfaces of human tumour and autologous human antitumour immunity in a small animal model.

Together, fully activated effector and exhausted-like human CD8^+^ T cells directly mediate anticancer activity. Our data support clinical exploration of activated and exhausted-like effector CD8^+^ T cell subsets alongside the recently more favoured stem-cell like phenotype for use in adoptive transfer and engineered T cell therapies.

### Limitations of the study

One limitation of the study is that transcriptomic profiling of T cells was only performed after *ex vivo* expansion. Transcriptomic analysis after *ex vivo* expansion allows for characterization of the infusion product that was adoptively transferred, similar to the infusion product of TIL therapy. Transcriptomic data before *ex vivo* expansion would be informative to extend the phenotyping of tumour-reactive T cells that was performed by flow cytometry.

Furthermore, sex as a biological variable was not explored in detail, which might unravel biological differences between males and females in the immune response to autologous tumours. This study was not designed to investigate sex-based differences, so the group size to perform analysis on subsets of animals was considered too small. In addition, the study planning did not consider baseline weight as a variable. However, post-hoc analysis on the effect of baseline weight on tumour volume did not show an effect of baseline weight on tumour volume, so the impact of baseline weight on the tumour volume was not explored in detail.

## Contributors

Conceptualization, J.M. and O.C.; Methodology, J.M., M.K. and O.C.; Investigation, J.M., M.K., L.E., L.O. and O.C.; Writing—Original Draft, J.M. and O.C.; Writing—Review & Editing, J.M. C.M. and O.C.; Funding Acquisition, O.C.; Resources, L.O. and C.M.; Supervision, O.C. J.M. and O.C. have accessed and verified the underlying data. All authors read and approved the final version of the manuscript.

## Data sharing statement

Supplemental data are provided with this paper. RNAseq data are available via AccessionID PRJEB75430 on ENA. All data are also available upon request.

## Declaration of interests

The authors declare that they have no competing interests.
