# Supplementary material for: Human effector CD8+ T cells with an activated and exhausted-like phenotype control tumour growth in vivo in a humanized tumour model
Source: eBioMedicine. 2024 Jul 9;106:105240. doi: 10.1016/j.ebiom.2024.105240 (PMC11296066; doi:10.1016/j.ebiom.2024.105240)
Supplement: Supplemental Figures [file mmc1.pdf]

Supplementary Material

Supplemental Figures 1-7

Human effector CD8$^+$ T cells with an activated and exhaustion-like phenotype control tumor growth *in vivo* in a humanized tumor model

Juliane Mietz[1], Meike Kaulfuss[1], Lukas Egli[1], Lennart Opitz[2], Christian Münz[3], Obinna Chijioke[1,4]

[1]Cellular Immunotherapy, Institute of Experimental Immunology, University of Zürich, Zürich, Switzerland
[2]Functional Genomics Center Zürich, University of Zürich/ETH Zürich, Zürich, Switzerland
[3]Viral Immunobiology, Institute of Experimental Immunology, University of Zürich, Zürich, Switzerland
[4]Institute of Medical Genetics and Pathology, University Hospital Basel, Basel, Switzerland

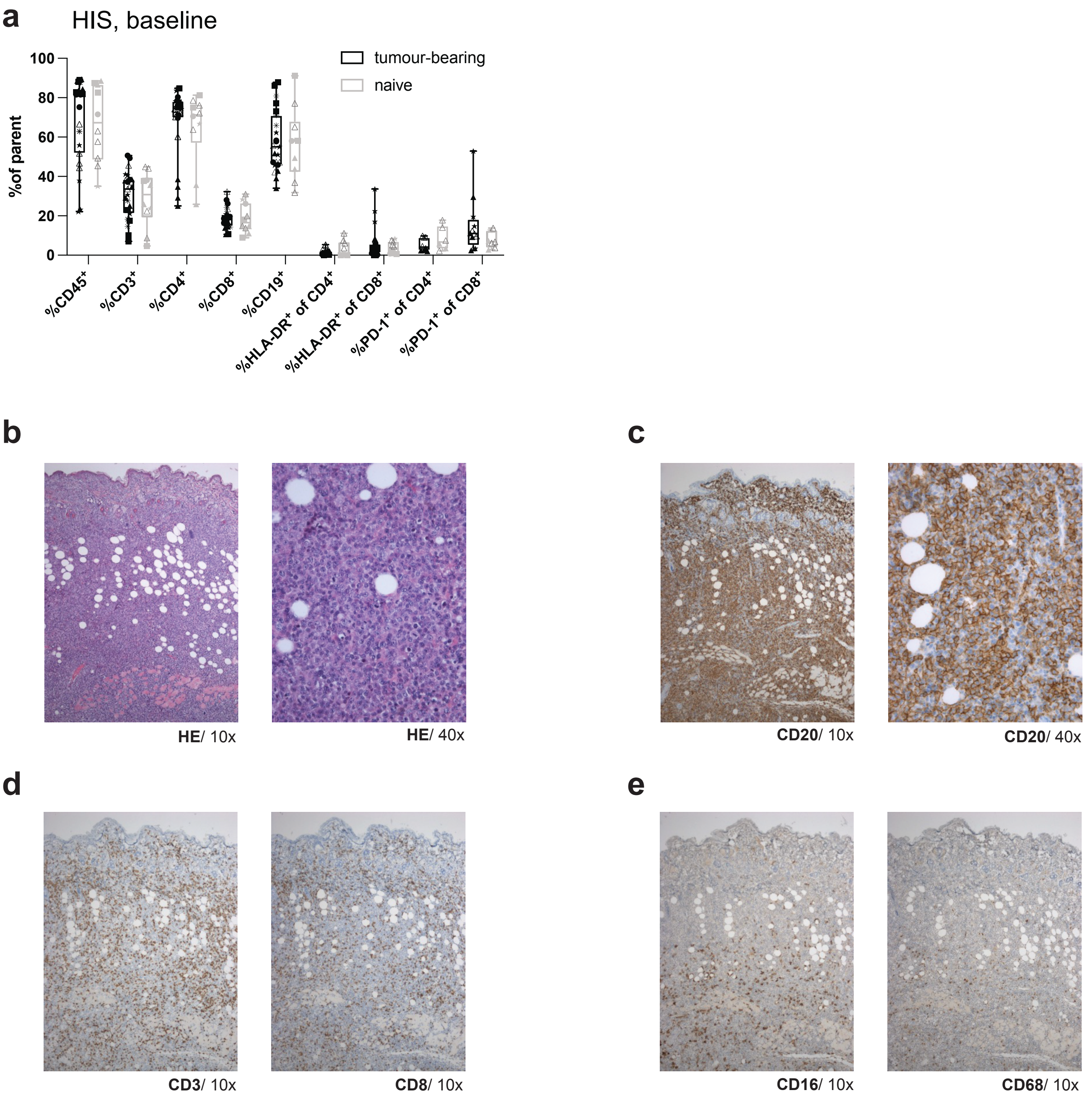
a
HIS, baseline
tumour-bearing
naive
%of parent
100
80
60
40
20
0
%CD45+
%CD3+
%CD4+
%CD8+
%CD19+
%HLA-DR+ of CD4+
%HLA-DR+ of CD8+
%PD-1+ of CD4+
%PD-1+ of CD8+
b
HE/ 10x
HE/ 40x
c
CD20/ 10x
CD20/ 40x
d
CD3/ 10x
CD8/ 10x
e
CD16/ 10x
CD68/ 10x

Supplemental Figure 1: Baseline human immune cell reconstitution and tumour infiltration by autologous human immune cells in HIS mice. a, baseline reconstitution of human immune cells in HIS mice before injection of tumours, analysed by flow cytometry**.** %CD45$^+$ is frequency within live lymphocytes. %CD3$^+$ and CD19$^+$ is frequency within %CD45$^+$; %CD4$^+$ and CD8$^+$ is frequency within CD3$^+$ T cells. (b-c), tumour histology by HE staining (**b**) and immunohistochemistry (IHC) showing CD20 expression of tumour cells (**c**). (d-e), human immune cell infiltration by IHC on tumour sections with staining for CD3 (left) and CD8 (right) (**d**) and CD16 (left) and CD68 (right) (**e**).

a, n(tumour bearing)=12-23, from 3-6 independent experiments; n(naïve)=7-10, from 3-6 independent experiments. Mixed-effects analysis with Šídák's multiple comparison test.

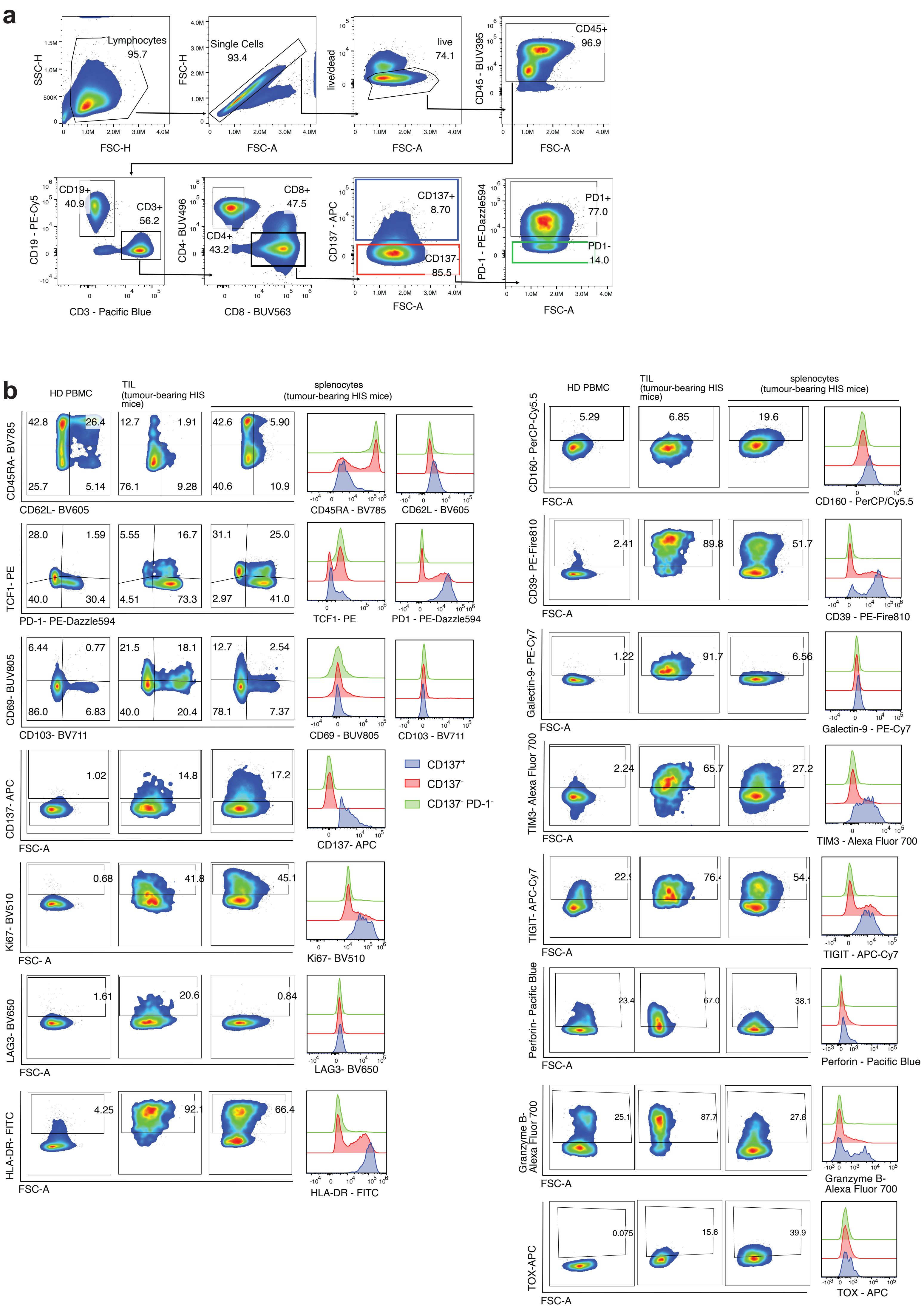
a
Lymphocytes
95.7
SSC-H
FSC-H
Single Cells
93.4
FSC-A
live/dead
live
74.1
CD45 - BUV395
CD45+
96.9
CD19 - PE-Cy5
CD19+
40.9
CD3+
56.2
CD3 - Pacific Blue
CD4- BUV496
CD8+
47.5
CD4+
43.2
CD8 - BUV563
CD137 - APC
CD137+
8.70
CD137-
85.5
PD-1 - PE-Dazzle594
PD1+
77.0
PD1-
14.0
b
HD PBMC
TIL
(tumour-bearing HIS mice)
splenocytes
(tumour-bearing HIS mice)
CD45RA- BV785
CD62L- BV605
CD45RA - BV785
CD62L - BV605
TCF1- PE
PD-1- PE-Dazzle594
TCF1- PE
PD1 - PE-Dazzle594
CD69- BUV805
CD103- BV711
CD69 - BUV805
CD103 - BV711
CD137- APC
FSC-A
CD137- APC
CD137+
CD137-
CD137- PD-1-
Ki67- BV510
FSC- A
Ki67- BV510
LAG3- BV650
LAG3- BV650
HLA-DR- FITC
HLA-DR - FITC
CD160- PerCP-Cy5.5
CD160 - PerCP/Cy5.5
CD39- PE-Fire810
CD39 - PE-Fire810
Galectin-9- PE-Cy7
Galectin-9 - PE-Cy7
TIM3- Alexa Fluor 700
TIM3 - Alexa Fluor 700
TIGIT- APC-Cy7
TIGIT - APC-Cy7
Perforin- Pacific Blue
Perforin - Pacific Blue
Granzyme B-
Alexa Fluor 700
TOX-APC
TOX - APC

Supplemental Figure 2: Additional flow cytometry data. **a**, representative flow cytometry plots showing gating strategy for $CD8^+$ T cells (thick black box), $CD137^+$ $CD8^+$ T cells (blue box), $CD137^-$ $CD8^+$ T cells (red box) or $CD137^-PD\text{-}1^-$ $CD8^+$ T cells (green box). **b**, representative flow cytometry plots from HD PBMC, TIL (tumour-bearing HIS mice) and splenocytes (tumour-bearing HIS mice) of indicated markers, gated on live human $CD8^+$ T cells. Representative histograms show fluorescence intensity of indicated markers of splenocytes gated on $CD137^+$ $CD8^+$, $CD137^-$ $CD8^+$ T cells or $CD137^-PD\text{-}1^-$ $CD8^+$ T cells. Histogram counts are normalized to mode. HD, healthy donor; HIS, NSG mice with reconstituted human immune system components, PBMC, peripheral blood mononuclear cells; TIL, tumour-infiltrating lymphocytes.

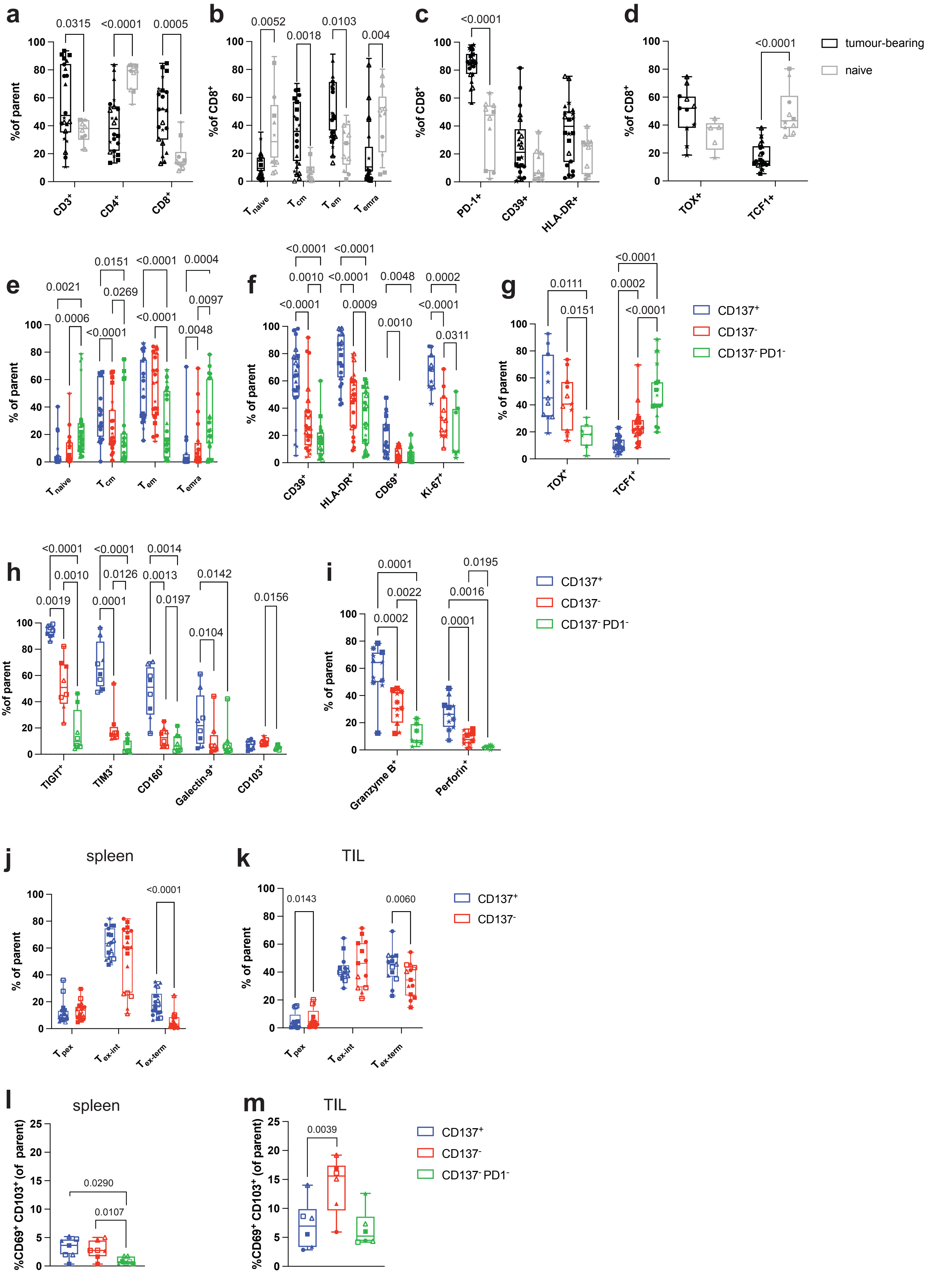
a
%of parent
0.0315
<0.0001
0.0005
CD3+
CD4+
CD8+
b
%of CD8+
0.0052
0.0018
0.0103
0.004
T naive
T cm
T em
T emra
c
%of CD8+
<0.0001
PD-1+
CD39+
HLA-DR+
d
%of CD8+
<0.0001
TOX+
TCF1+
tumour-bearing
naive
e
% of parent
0.0021
0.0006
0.0151
0.0269
<0.0001
<0.0001
<0.0001
0.0004
0.0097
0.0048
T naive
T cm
T em
T emra
f
% of parent
<0.0001
<0.0001
0.0010
<0.0001
<0.0001
0.0009
0.0048
0.0010
0.0002
<0.0001
0.0311
CD39+
HLA-DR+
CD69+
Ki-67+
g
% of parent
0.0111
0.0151
0.0002
<0.0001
<0.0001
TOX+
TCF1+
CD137+
CD137-
CD137- PD1-
h
%of parent
<0.0001
0.0010
0.0019
<0.0001
0.0126
0.0001
0.0014
0.0013
0.0197
0.0142
0.0104
0.0156
TIGIT+
TIM3+
CD160+
Galectin-9+
CD103+
i
% of parent
0.0001
0.0022
0.0002
0.0195
0.0016
0.0001
Granzyme B+
Perforin+
CD137+
CD137-
CD137- PD1-
j
spleen
% of parent
<0.0001
T pex
T ex-int
T ex-term
k
TIL
% of parent
0.0143
0.0060
T pex
T ex-int
T ex-term
CD137+
CD137-
l
spleen
%CD69+ CD103+ (of parent)
0.0290
0.0107
m
TIL
%CD69+ CD103+ (of parent)
0.0039
CD137+
CD137-
CD137- PD1-

Supplemental Figure 3: T cell profiling in peripheral blood, spleen and tumour of tumour-bearing HIS mice. PBMC, splenocytes and TIL were isolated from tumour-bearing HIS mice and analysed by flow cytometry. **a,** frequency of T cells in peripheral blood of tumour-bearing or non-tumour bearing HIS mice (naïve); parent population refers to frequency (%) of $CD3^+$ T cells within human $CD45^+$ cells and $CD4^+$ and $CD8^+$ T cells within $CD3^+$ T cells. **b**, $CD8^+$ T cell differentiation defined as $T_{naïve}$ ($CD45RA^+CD62L^+$), T_{CM} ($CD45RA^-CD62L^+$), T_{EM} ($CD45RA^-CD62L^-$), T_{EMRA} ($CD45RA^+CD62L^-$) in tumour-bearing or naïve HIS mice. **c-d**, expression of indicated markers on human $CD8^+$ T cells in peripheral blood of tumour-bearing or naïve HIS mice. **e-i**, proportion of $CD8^+$ T cell subsets and expression of individual markers in spleen of tumour-bearing HIS mice within $CD137^+$, $CD137^-$ or $CD137^-PD\text{-}1^-$ populations. (j-k), distribution of T_{pex} ($TCF1^+PD\text{-}1^+$), $T_{ex\text{-}int}$ ($TCF1^-PD\text{-}1^+CD69^-$) and $T_{ex\text{-}term}$ ($TCF1^-PD\text{-}1^+CD69^+$) subsets within $CD137^+$ or $CD137^-$ populations from spleen (**j**) or tumour (TIL) (**k**) of tumour-bearing HIS mice. (l-m), T_{RM}-like cells ($CD69^+CD103^+$) within $CD137^+$ or $CD137^-$ $CD8^+$ T cell populations in spleen (**l**) or tumour (TIL) (**m**) of tumour-bearing HIS mice.

a-c, n(tumour bearing)=23, n(naïve)=10 from 6 independent experiments. 2way ANOVA with Šídák's multiple comparison test. d, n(tumour bearing)=10-23, n(naïve)=5-10 from 2-6 independent experiments. 2way ANOVA with Šídák's multiple comparison test. e, j, n=23 from 6 independent experiments. 2way RM ANOVA with Tukey's multiple comparison test. f-g, n=7-23, from 2-6 independent experiments. Mixed-effects analysis with matching and Tukey's multiple comparison test. h, n=8, from 3 independent experiments, 2way RM ANOVA with Tukey's multiple comparison test. i, n=7-11, from 2-3 independent experiments. Mixed-effects analysis with matching and Tukey's multiple comparison test. k, n=13, from 4 independent experiments. 2way RM ANOVA with Tukey's multiple comparison test. l-m, n=7 from 3 independent experiments. 2way RM ANOVA with Tukey's multiple comparison test. For each experiment, a different HPC donor was used for HIS mouse reconstitution and generation of autologous tumour. Data from individual experiments are indicated by different symbols, with individual mice from the same experiment indicated by the same symbol.

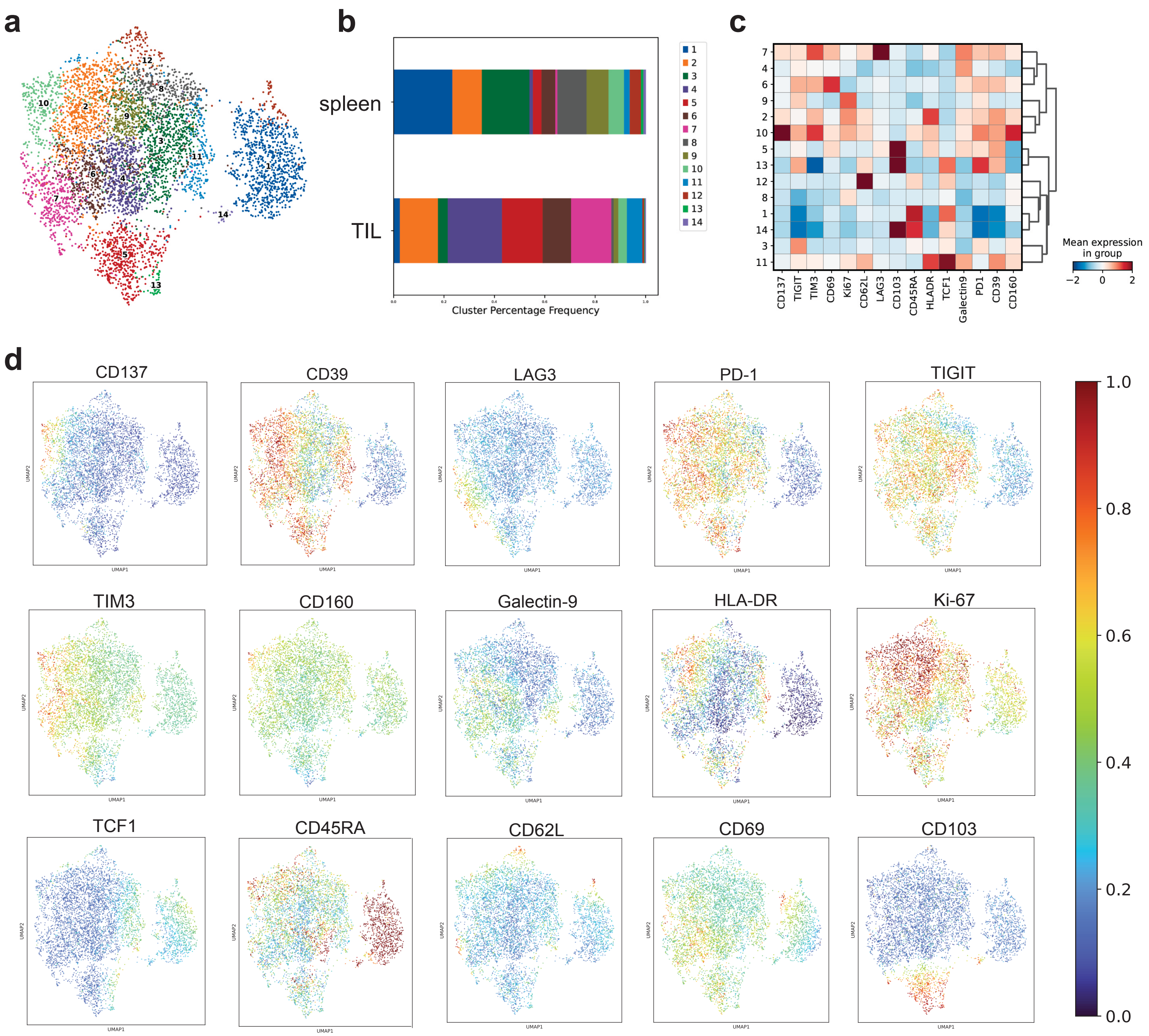
a
b
spleen
TIL
Cluster Percentage Frequency
c
Mean expression in group
CD137
TIGIT
TIM3
CD69
Ki67
CD62L
LAG3
CD103
CD45RA
HLADR
TCF1
Galectin9
PD1
CD39
CD160
d
CD137
CD39
LAG3
PD-1
TIGIT
TIM3
CD160
Galectin-9
HLA-DR
Ki-67
TCF1
CD45RA
CD62L
CD69
CD103

Supplemental Figure 4: Co-expression analysis of CD8$^+$ T cells in tumour-bearing HIS mice. Splenocytes and TIL were isolated from tumour-bearing HIS mice and CD8$^+$ T cells analysed by flow cytometry. **a**, cluster visualization by UMAP of CD8$^+$ T cells from spleen and TIL. **b**, bar chart showing distribution of CD8$^+$ T cell clusters in spleen and TIL. **c**, heatmap depicting mean expression of individual markers in the respective CD8$^+$ T cell clusters. **d**, UMAP plots showing expression of individual markers. Data are pooled from 4 independent experiments, n=6-8 per group. For each experiment, a different HPC donor was used for HIS mouse reconstitution and generation of autologous tumour.

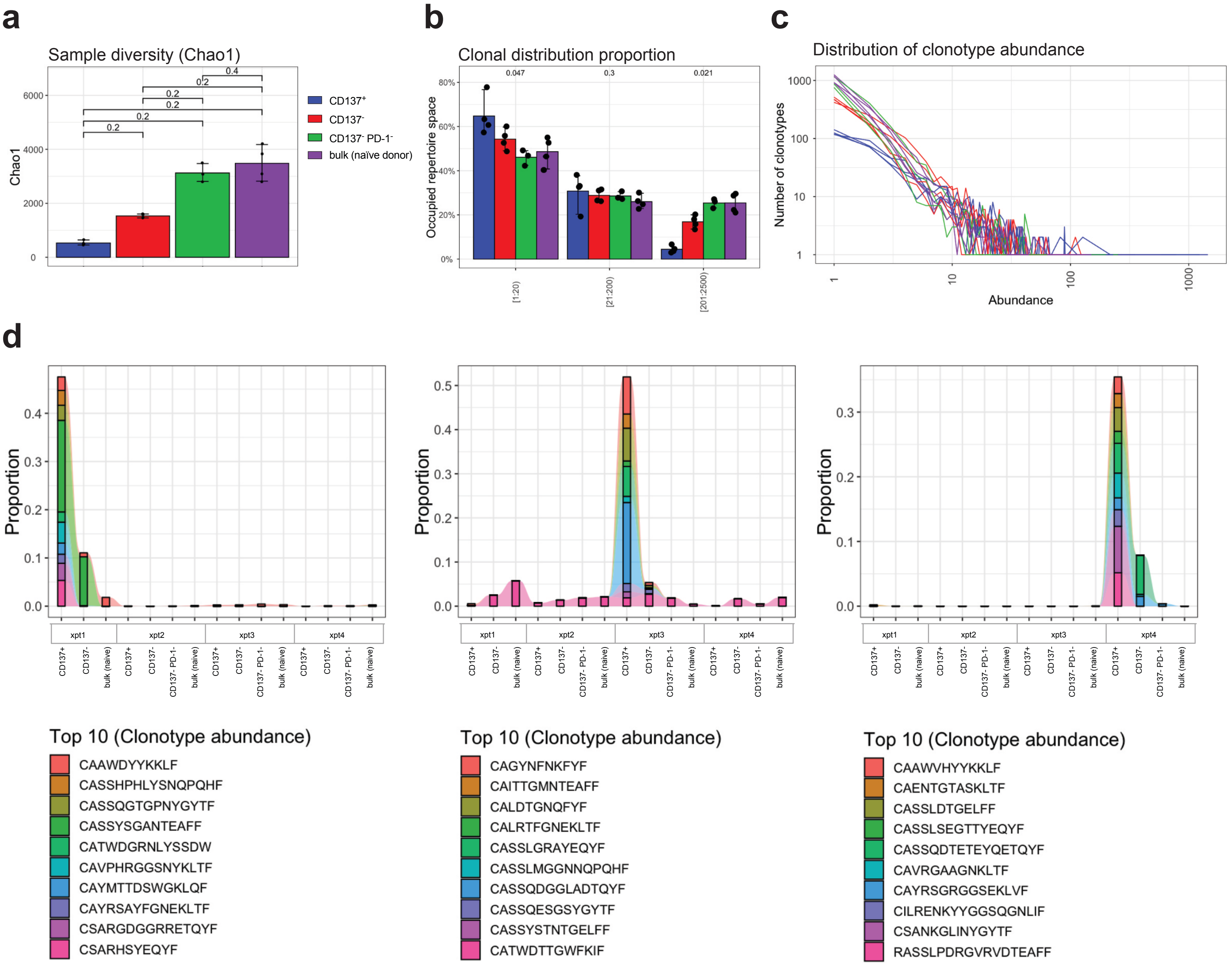
a
Sample diversity (Chao1)
Chao1
0
2000
4000
6000
0.2
0.2
0.2
0.2
0.2
0.4
CD137+
CD137-
CD137- PD-1-
bulk (naïve donor)
b
Clonal distribution proportion
Occupied repertoire space
0%
20%
40%
60%
80%
0.047
0.3
0.021
[1:20)
[21:200)
[201:2500)
c
Distribution of clonotype abundance
Number of clonotypes
1
10
100
1000
Abundance
d
Proportion
xpt1
xpt2
xpt3
xpt4
CD137+
CD137-
bulk (naive)
CD137- PD-1-
Top 10 (Clonotype abundance)
CAAWDYYKKLF
CASSHPHLYSNQPQHF
CASSQGTGPNYGYTF
CASSYSGANTEAFF
CATWDGRNLYSSDW
CAVPHRGGSNYKLTF
CAYMTTDSWGKLQF
CAYRSAYFGNEKLTF
CSARGDGGRRETQYF
CSARHSYEQYF
Top 10 (Clonotype abundance)
CAGYNFNKFYF
CAITTGMNTEAFF
CALDTGNQFYF
CALRTFGNEKLTF
CASSLGRAYEQYF
CASSLMGGNNQPQHF
CASSQDGGLADTQYF
CASSQESGSGYGYTF
CASSYSTNTGELFF
CATWDTTGWFKIF
Top 10 (Clonotype abundance)
CAAWVHYYKKLF
CAENTGTASKLTF
CASSLDTGELFF
CASSLSEGTTYEQYF
CASSQDTETEYQETQYF
CAVRGAAGNKLTF
CAYRSGRGGSEKLVF
CILRENKYYGGSQGNLIF
CSANKGLINYGYTF
RASSLPDRGVRVDTEAFF

Supplemental Figure 5: Additional TCR repertoire analyses including clonotype tracking of human tumour-reactive CD137^{+} CD8^{+} T cells. a, TCR sequence (CDR3 of *TRA*, *TRB*, *TRG* and *TRD*) sample diversity estimation using Chao1 method. Kruskal-Wallis test with Holm-Bonferroni correction. **b,** clonal proportion distribution of clonotypes with the indicated indices on the x-axis (e.g. [1:20) contains the 20 largest clonotypes). **c**, distribution of clonotype abundance. **d,** tracking of clonotypes over populations and experiments/donors. The top 10 most abundant clonotypes of the TCR repertoire of the CD137^{+} population from 3 independent experiments are shown (each experiment with different human HPC donor for reconstitution of HIS mice and autologous tumour).

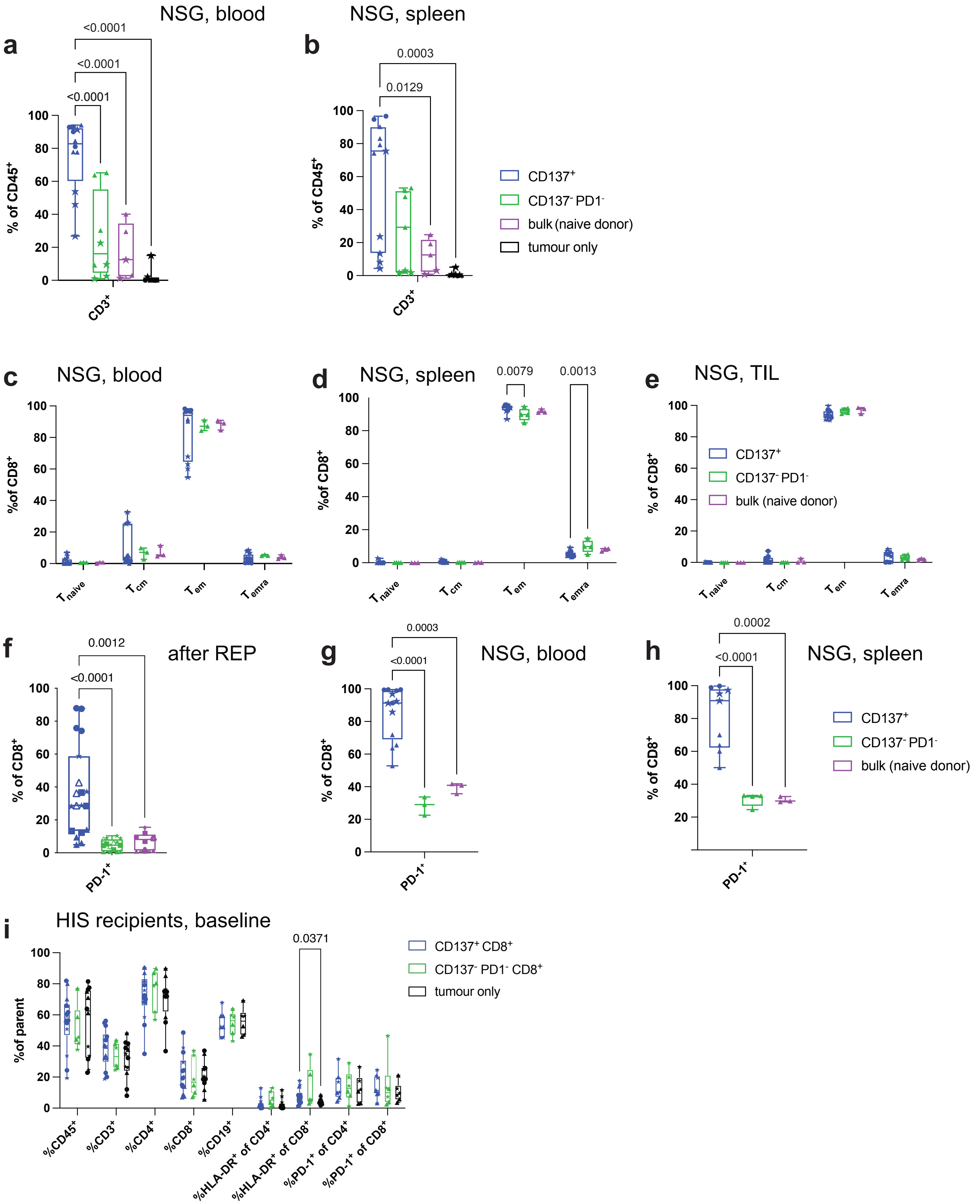
a
NSG, blood
% of CD45+
CD3+
<0.0001
<0.0001
<0.0001
b
NSG, spleen
% of CD45+
CD3+
0.0003
0.0129
CD137+
CD137- PD1-
bulk (naive donor)
tumour only
c
NSG, blood
%of CD8+
T naive
T cm
T em
T emra
d
NSG, spleen
%of CD8+
0.0079
0.0013
T naive
T cm
T em
T emra
e
NSG, TIL
%of CD8+
CD137+
CD137- PD1-
bulk (naive donor)
T naive
T cm
T em
T emra
f
after REP
% of CD8+
PD-1+
0.0012
<0.0001
g
NSG, blood
% of CD8+
PD-1+
0.0003
<0.0001
h
NSG, spleen
% of CD8+
PD-1+
0.0002
<0.0001
CD137+
CD137- PD1-
bulk (naive donor)
i
HIS recipients, baseline
%of parent
0.0371
CD137+ CD8+
CD137- PD1- CD8+
tumour only
%CD45+
%CD3+
%CD4+
%CD8+
%CD19+
%HLA-DR+ of CD4+
%HLA-DR+ of CD8+
%PD-1+ of CD4+
%PD-1+ of CD8+

Supplemental Figure 6: T cell distribution, differentiation and PD-1 expression on CD8$^+$ T cells in tumour-bearing NSG recipients after ACT of CD137$^+$ CD8$^+$ T cells and baseline reconstitution of HIS recipients. (a-b), frequency of CD3$^+$ T cells (% of human CD45$^+$ cells) at sacrifice in blood (**a**) or spleen (**b**) of tumour-bearing NSG mice after ACT of the indicated cell populations. (c-e), CD8$^+$ T cell differentiation in blood (**c**), spleen (**d**) or TIL (**e**) of tumour-bearing NSG mice after ACT of indicated cell populations. **f**, frequency of PD-1 expression after *ex vivo* expansion of the indicated cell populations. (g-h), frequency of PD-1 expression in blood (**g**) or spleen (**h**) of tumour-bearing NSG mice after ACT of indicated cell populations. **i**, baseline reconstitution of human immune cells in HIS recipient mice before injection of tumours and adoptive cell transfer, analysed by flow cytometry. a,b, n=5-12, from 2-3 independent experiments. Only data points in which >100 CD45$^+$ events were recorded are shown. One-way ANOVA with Tukey's multiple comparison test. c-e, g-h, n=3-13, from 1-3 independent experiments. Only data points in which >100 CD8$^+$ events were recorded are shown. One-way ANOVA with Tukey's multiple comparison test. f, n=8-23, from 4-6 independent experiments. Kruskal-Wallis test with Dunn's multiple comparison test. i, n=6-14, from 2-3 independent experiments. Mixed-effects analysis with Šídák's multiple comparison test. %CD45$^+$ is frequency within live lymphocytes. %CD3$^+$ and CD19$^+$ is frequency within %CD45$^+$; %CD4$^+$ and CD8$^+$ is frequency within CD3$^+$ T cells. For each experiment, a different HPC donor was used for HIS mouse reconstitution and generation of autologous tumour. Data from individual experiments are indicated by different symbols, with individual mice from the same experiment indicated by the same symbol.

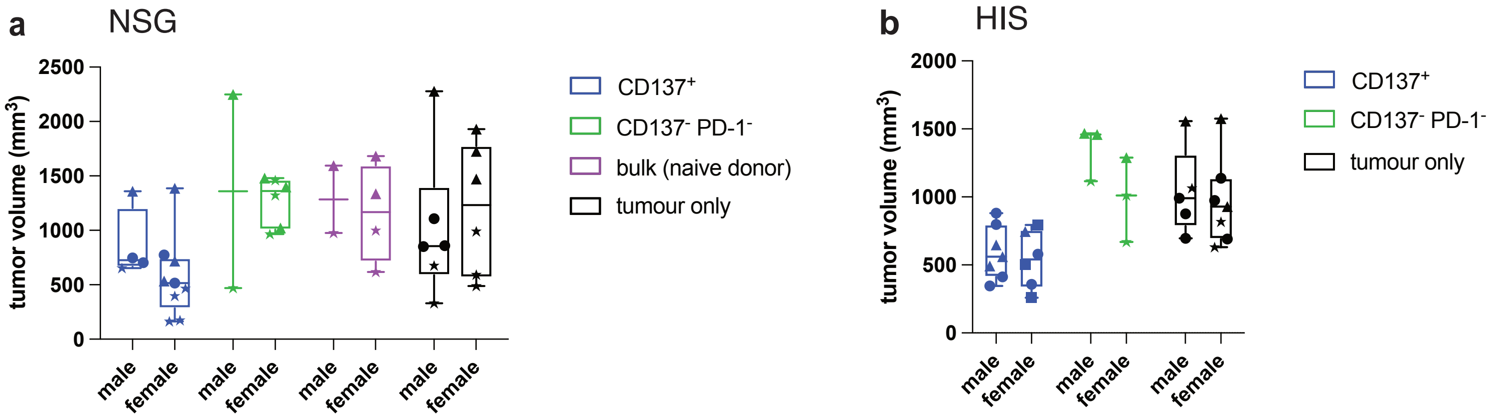
a
NSG
tumor volume (mm3)
2500
2000
1500
1000
500
0
male
female
male
female
male
female
male
female
CD137+
CD137- PD-1-
bulk (naive donor)
tumour only
b
HIS
tumor volume (mm3)
2000
1500
1000
500
0
male
female
male
female
male
female
CD137+
CD137- PD-1-
tumour only

Supplemental Figure 7: Sex-based post hoc analysis of tumour volume after adoptive T cell transfer. (a-b), tumour volume of male and female (**a**) NSG or (**b**) HIS recipient mice on day of sacrifice after adoptive transfer of the indicated T cell populations. 2way ANOVA with Šídák's multiple comparisons test in which male versus female was compared within treatment groups. Data are pooled from 2-3 independent experiments. a, n($CD137^{+}$, male)=4, n($CD137^{+}$, female)=9, n($CD137^{-}PD\text{-}1^{-}$, male)=2, n($CD137^{-}PD\text{-}1^{-}$, female)=6, n(bulk, male)=2, n(bulk, female)=4, n(tumour only, male)=6, n(tumour only, female)=6. b, n($CD137^{+}$, male)=7, n($CD137^{+}$, female)=6, n($CD137^{-}PD\text{-}1^{-}$, male)=3, n($CD137^{-}PD\text{-}1^{-}$, female)=3, n(tumour only, male)=5, n(tumour only, female)=7.
